# Functional Loss of Two Ceramide Synthases Elicits Autophagy-Dependent Lifespan Extension in *C. elegans*

**DOI:** 10.1371/journal.pone.0070087

**Published:** 2013-07-19

**Authors:** Mai-Britt Mosbech, Rikke Kruse, Eva Bang Harvald, Anne Sofie Braun Olsen, Sandra Fernandez Gallego, Hans Kristian Hannibal-Bach, Christer S. Ejsing, Nils J. Færgeman

**Affiliations:** Department of Biochemistry and Molecular Biology, University of Southern Denmark, Odense, Denmark; Rutgers New Jersey Medical School, United States of America

## Abstract

Ceramide and its metabolites constitute a diverse group of lipids, which play important roles as structural entities of biological membranes as well as regulators of cellular growth, differentiation, and development. The *C. elegans* genome comprises three ceramide synthase genes; *hyl-1, hyl-2,* and *lagr-1*. HYL-1 function is required for synthesis of ceramides and sphingolipids containing very long acyl-chains (≥C24), while HYL-2 is required for synthesis of ceramides and sphingolipids containing shorter acyl-chains (≤C22). Here we show that functional loss of HYL-2 decreases lifespan, while loss of HYL-1 or LAGR-1 does not affect lifespan. We show that loss of HYL-1 and LAGR-1 functions extend lifespan in an autophagy-dependent manner, as knock down of the autophagy-associated gene ATG-12 abolishes *hyl-1;lagr-1* longevity. The transcription factors PHA-4/FOXA, DAF-16/FOXO, and SKN-1 are also required for the observed lifespan extension, as well as the increased number of autophagosomes in *hyl-1;lagr-1* animals. Both autophagic events and the transcription factors PHA-4/FOXA, DAF-16, and SKN-1 have previously been associated with dietary restriction-induced longevity. Accordingly, we find that *hyl-1;lagr-1* animals display reduced feeding, increased resistance to heat, and reduced reproduction. Collectively, our data suggest that specific sphingolipids produced by different ceramide synthases have opposing roles in determination of *C. elegans* lifespan. We propose that loss of HYL-1 and LAGR-1 result in dietary restriction-induced autophagy and consequently prolonged longevity.

## Introduction

Besides being required for the integrity of cellular membranes, sphingolipids, and in particular ceramide and sphingosine-1-phosphate, have emerged as bioactive signalling molecules involved in regulation of cell growth, differentiation, senescence, and apoptosis [1]–[3]. Ceramide is at the central hub of sphingolipid metabolism and is the precursor for complex sphingolipids such as sphingomyelin and glycosphingolipids. Sphingosine-based ceramide species are generated from dihydroceramide in a desaturation step that introduces a 4,5 double bond in the sphingoid base, which constitutes the backbone of all sphingolipids. Ceramide can also be deacylated to sphingosine, which can be phosphorylated by sphingosine kinase to sphingosine-1-phosphate. Synthesis of sphingosine-1-phosphate constitutes the only exit-route from the sphingolipid pathway by the action of sphingosine-1-phosphate lyase, yielding ethanolamine phosphate and hexadecanal, which can be utilized for production of various other lipids. Ceramide is synthesized *de novo* from palmitate and serine, which through a series of reactions is converted to dihydrosphingosine, which again is acylated to yield dihydroceramide by the action of ceramide synthases. The complexity of sphingolipid metabolism and the biological functions it affects are vast. Each class of sphingolipid has been generally thought of as entities, being regulated and acting in the same way, however, by virtue of their structural diversities each individual molecular sphingolipid species may have distinct regulatory functions in specific cellular pathways. Mammals contain six ceramide synthases, CERS1-6 (formerly named Lass1-6), which are all differentially expressed and show substrate specificity towards subsets of fatty acyl-CoAs, characterized by chain length and degree of saturation and hydroxylation (reviewed in [4]). The fact that targeted knock-down of each *CERS* results in increased mRNA levels of non-targeted *CERS* [5], emphasizes the importance of these enzymes in cellular homeostasis.

Several model organisms have been utilized to address sphingolipid metabolism, each offering different advantages. In *C. elegans* the sphingolipids are of less structural complexity as the sphingoid long chain base exclusively constitutes a C17 *iso*- or *anteiso*-branched chain as illustrated in Figure S1. *C. elegans* comprises three ceramide synthases HYL-1, HYL-2, and LAGR-1, each containing a Lag1p motif required for ceramide synthase activity [6], [7]. It has been shown that radiation-induced apoptosis in the germ line is inhibited in *hyl-1(ok976)*, *lagr-1(gk327/gk331)*, and *hyl-1(ok976);lagr-1(gk327)* animals, which can be relieved by injection of C16 ceramide [8]. Moreover, mutants of sphingosine kinase-1 (*sphk-1(ok1097))* have an elevated germ line death baseline compared to wild type worms and are hypersensitive to radiation-induced apoptosis, effects which are not observed in *lagr-1(gk327);sphk-1(ok1097)* animals [8]. These observations collectively suggest that specific sphingolipids originating from HYL-1 and/or LAGR-1 ceramide production are involved in germ line radiation-induced apoptosis. It has also been reported that worms lacking ceramide glucosyltransferases (CGTs) arrest at the first larval stage and that this arrest can be rescued by CGT expression in the most anterior and posterior intestinal cells, implying that lack of glycosphingolipids results in starvation-induced growth arrest by impaired feeding [9]. Menuz et al. have recently shown that *hyl-2(gnv1/tm2031)* animals are sensitive to anoxia while *hyl-1(gk203/ok976)* animals are more resistant [10]. They also showed that C20-22 ceramides and sphingomyelins are more abundant in *hyl-1(ok976)* animals while C24-26 ceramides are less abundant. In contrast, *hyl-2(gnv1)* animals contain less C20-22 ceramides and sphingomyelins but more with C24-26. Tedesco et al. [7] recently showed that RNAi targeted against the Lag1p motif in *hyl-1* extended lifespan and reduced mRNA levels of both *hyl-1* and *hyl-2*, while *hyl-1* deletion mutants (*hyl-1(ok976/gk203))* showed no alterations in lifespan and an increased level of *hyl-2* mRNA.

In the present study we have further examined the role of ceramide synthases in longevity and find that simultaneous deletion of *hyl-1* and *lagr-1* results in a lifespan extension, which depends on autophagy and the transcriptions factors PHA-4, DAF-16, and SKN-1. We also find that *hyl-1;lagr-1* animals have an altered sphingolipid profile and that the extended lifespan can be normalized by *sphk-1* RNAi, indicating the composition and/or levels of certain sphingolipids are key effectors in the lifespan extension.

## Results

### The Autophagy-associated Gene ATG-12, and the Transcription Factors PHA-4, DAF-16, and SKN-1 are Required for *hyl-1;lagr-1* Longevity

**Figure 1:**
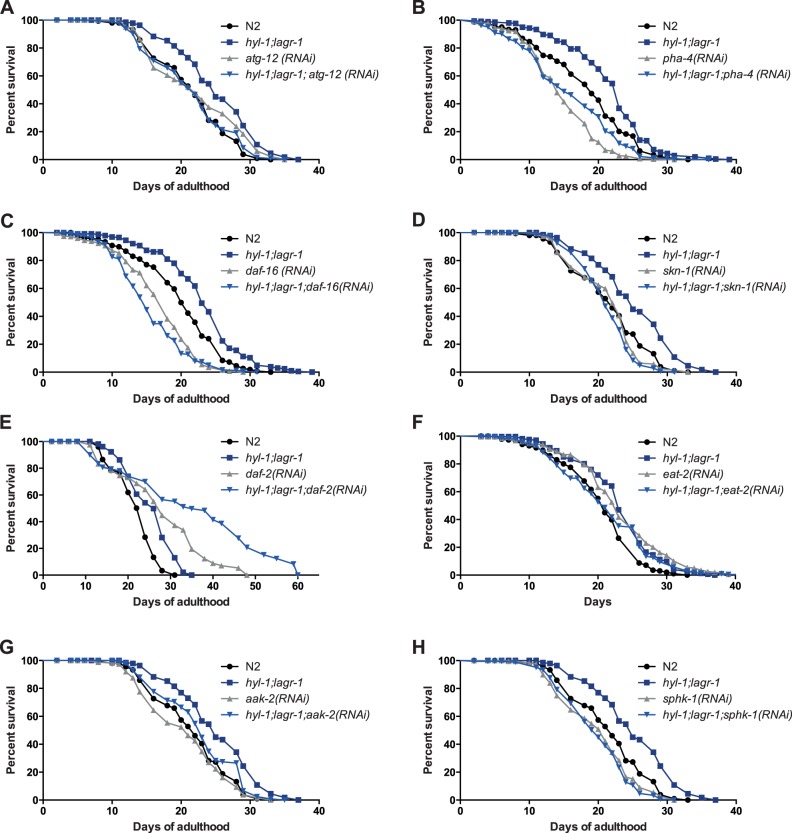
Knock-down of PHA-4, DAF-16, SKN-1, ATG-12, or SPHK-1 affect the extended longevity of *hyl-1;lagr-1.* Cumulative survival curves of N2 and *hyl-1;lagr-1* worms grown at 20°C subjected to either empty vector control bacteria (L4440) or the indicated RNAi from the early adult stage. (A) When subjected to *atg-12* RNAi, the extended lifespan of *hyl-1;lagr-1* is normalized to the extent of *atg-12(RNAi)* control animals, P = 0.3053. (B) When subjected to *pha-*4 RNAi, the extended lifespan of *hyl-1;lagr-1* is normalized to the extent of *pha-4(RNAi)* control animals, P = 0.2369. (C) When subjected to *daf-16* RNAi, the extended lifespan of *hyl-1;lagr-1* is decreased beyond the extent of *daf-16(RNAi)* control animals, P = 0.0002. (D) When subjected to *skn-1* RNAi, the extended lifespan of *hyl-1;lagr-1* is normalized to the extent of *skn-1(RNAi)* control animals, P = 0.5476. (E) When subjected to *daf-2* RNAi, *hyl-1;lagr-1* lifespan is further extended compared to both *hyl-1;lagr-1* control animals, P<0.0001, and *daf-2(RNAi)* control animals, P<0.0001. (F) When subjected to *eat-2* RNAi, *hyl-1;lagr-1* lifespan is decreased compared to *hyl-1;lagr-1* control animals, P = 0.0002, while the lifespan of *eat-2(RNAi)* animals is extended compared to wild-type control animals, P<0.0001. (G) When subjected to *aak-2* RNAi, *hyl-1;lagr-1* lifespan is decreased compared to *hyl-1;lagr-1* animals, P = 0.0009, while no lifespan effect is seen when comparing *aak-2(RNAi)* animals to wild-type control animals, P = 0.0975. (H) When subjected to *sphk-1* RNAi, the extended lifespan of *hyl-1;lagr-1* is normalized to the extent of *sphk-1(RNAi)* control animals, P = 0.8002. For additional details about these experiments, see Table 1.

To address the role of ceramide synthases in *C. elegans* lifespan we obtained strains carrying a loss of function allele of each of the genes encoding a ceramide synthase. After backcrossing we made all possible combinations of ceramide synthase mutant strains, resulting in a collection of ceramide synthase mutants; *hyl-1(ok976), hyl-2(ok1766), lagr-1(gk331), hyl-1;lagr-1(ok976;gk331),* and *hyl-2;lagr-1 (ok1766;gk331)*. In accordance with previous findings, we found that *hyl-1;hyl-2* mutants are unviable and cannot be obtained by genetic crossing [10]. We first examined the lifespan of each of these strains under standard culture conditions. In contrast to previous observations [10], we found that *hyl-2* and *hyl-2;lagr-1* animals live significantly shorter than wild type animals (Figure S2), while *hyl-1;lagr-1* animals live significantly longer compared to wild type animals (Figure 1 and Figure S2). In an effort to identify the mechanism(s) underlying the *hyl-1;lagr-1* lifespan extension, we asked whether functional loss of HYL-1 and LAGR-1 affected the expression of key longevity genes including the insulin receptor *daf-2*, the transcription factors *daf-16*, *pha-4,* and *skn-1*, the autophagy genes *atg-3*, *atg-12*, *atg-18,* and *lgg-1* as well as the catalytic subunit of the AMP-dependent kinase *aak-2*. Among the genes examined, we only found a small, yet significant increase in *pha-4* expression, while *atg-12* expression was significantly reduced in *hyl-1;lagr-1* animals (Figure S3). Despite the small differences in their expression level, we hypothesized that these factors could be involved in the life span extension of *hyl-1;lagr-1* animals, and thus knocked down these longevity genes by RNAi. Consistently, *hyl-1;lagr-1* animals lived significantly longer than wild type animals when fed empty vector control bacteria (Figure 1, Table 1, P<0.0001). Autophagy is a recycling process, which is induced during stress conditions, and has been shown to be required for the lifespan extension induced by decreased insulin/insulin-like growth factor-1 signaling (IIS) [11] or dietary restriction (DR) [12], [13]. We found that the extended lifespan of *hyl-1;lagr-1* depends on autophagy, as knock down of *atg-12* normalized longevity (Figure 1A, P = 0.3053). Depending on how dietary restriction is induced [14], DR-induced longevity depends on the transcription factors PHA-4/FOXA [13], [15] as well as the transcription factor SKN-1 [16]. Knock down of *pha-4* and *skn-1* decreased the lifespan of *hyl-1;lagr-1* animals to that of *pha-4(RNAi)* and *skn-1(RNAi)* N2 animals (Figure 1B, P = 0.2369 and Figure 1D, P = 0.5476), implying that *hyl-1;lagr-1* animals are dietary restricted. Mutations in *eat-2*, encoding a subunit of the nicotinic acetylcholine receptor, results in impaired pharyngeal pumping and defecation. *eat-2* mutants are therefore often used as a genetic surrogate to study dietary induced lifespan extension [17]. Since knock down of *eat-2* only increases lifespan in wild type animals and not in *hyl-1;lagr-1* animals (Figure 1F) it supports the notion that *hyl-1;lagr-1* animals are dietary restricted. Compromised IIS increases longevity in a FOXO/DAF-16 dependent manner [18] and interestingly, we found that knock down of *daf-16* decreases the lifespan of *hyl-1;lagr-1* beyond that of *daf-16(RNAi)* N2 animals (Figure 1C, P = 0.0002). Despite the fact that the lifespan extension of *daf-2* animals depends on DAF-16 function, the increased level of autophagy in *daf-2* animals has been shown to be independent of both DAF-16 and PHA-4, implying that autophagy on its own is not sufficient to extend lifespan and led to the suggestion that the role of DAF-16 is to orchestrate proper utilization of components released from autophagic events [15]. These observations prompted us to address whether knockdown of *daf-2* affected *hyl-1;lagr-1* longevity, and found that the lifespan of *hyl-1;lagr-1* animals is further extended by *daf-2* RNAi compared to both *hyl-1;lagr-1* fed control bacteria and *daf-2(RNAi)* N2 animals (Figure 1E, P *= *0.0002 and P<0.0001, respectively). These findings indicate that *hyl-1;lagr-1* longevity is elicited independently of IIS and that *hyl-1;lagr-1* animals are more susceptibility to functional loss of DAF-16. We also found that the life span extension of *hyl-1;lagr-1* animals is independent of the AMP-dependent kinase AMPK, as knock down of *aak-2*, the catalytic subunit of AMPK [19], did not abolish the longevity phenotype of the dobbelt mutant (Figure 1G). Interestingly, we found that knock down of *sphk-1* decreases *hyl-1;lagr-1* lifespan to the same level as *sphk-1(RNAi)* N2 animals (Figure 1H, P = 0.8002), indicating that the turn-over of sphingolipids or specific sphingosine-1-phosphate(s) regulate the longevity response.

**Table 1:** Adult lifespan of *hyl-1;lagr-1* and N2 control worms subjected to empty vector control or the indicated RNAi at 20°C.

Strain	Adult-only RNAi treatment	RNAi lifespan^a^(days)	Number of animals^b^ (trials)	Control lifespan^c^ (days)	Number of control animals^b^ (trials)	Lifespan change (%)	p-value vs. control^d^
N2	*pha-4*	**14**/14.4	291/360 (3)	**19**/17.6	293/360 (3)	−26.3	**<0.0001**
	*daf-16*	**18**/16.2	280/360 (3)	**20**/19.5	279/360 (3)	−10.0	**<0.0001**
	*skn-1*	**22**/21.2	156/192 (2)	**22**/21.0	112/192 (2)	0	0.8563
	*atg-12*	**22**/22.2	135/192 (2)	**22**/21.0	112/192 (2)	0	0.5426
	*sphk-1*	**21**/19.6	157/192 (2)	**22**/21.0	112/192 (2)	−4.5	0.014
	*aak-2*	**21**/20.2	143/192 (2)	**22**/21.0	112/192 (2)	−4.5	0.0975
	*daf-2*	**28/**26.4	67/96 (1)	**24/**21.6	63/96 (1)	27.3	**<0.0001**
	*eat-2*	**23**/22.1	243/360 (2)	**21**/20.2	200/240 (2)	9.5	**<0.0001**
*hyl-1;lagr-1*	*pha-4*	**15**/14.8	277/348 (3)	**23**/20.6	239/351 (3)	−34.8	**<0.0001**
	*daf-16*	**15**/15.0	304/360 (3)	**23**/22.4	226/360 (3)	−34.8	**<0.0001**
	*skn-1*	**21**/21.0	146/192 (2)	**25**/24.8	122/192 (2)	−16.0	**<0.0001**
	*atg-12*	**22**/24.8	138/192 (2)	**25**/24.8	122/192 (2)	−12.0	**<0.0001**
	*sphk-1*	**20**/21.1	152/192 (2)	**25**/24.8	122/192 (2)	−20.0	**<0.0001**
	*aak-2*	**23**/21.7	128/192 (2)	**25**/24.8	122/192 (2)	−8.0	**0.0009**
	*daf-2*	**35/**34.9	74/96 (1)	**26/**25.2	47/96 (1)	34.6	**0.0089**
	*eat-2*	**21**/20.1	257/358 (2)	**23**/22.6	159/240 (2)	−8.7	**0.0002**
*hyl-1:lagr-1* vs. N2	control	**23**/20.6	239/351 (3)	**19**/17.6	293/360 (3)	21.1	**<0.0001**
	*pha-4*	**15**/14.8	277/348 (3)	**14**/14.4	291/360 (3)	7.1	0.2369
	control	**23**/22.4	226/360 (3)	**20**/19.5	279/360 (3)	15.0	**<0.0001**
	*daf-16*	**15**/15.0	304/360 (3)	**18**/16.2	280/360 (3)	−16.7	**0.0002**
	control	**25**/24.8	112/192 (2)	**22**/21.0	122/192 (2)	13.6	**<0.0001**
	*skn-1*	**21**/21.0	146/192 (2)	**22**/21.2	156/192 (2)	−4.5	0.5476
	*atg-12*	**22**/24.8	138/192 (2)	**22**/22.2	135/192 (2)	0	0.3053
	*sphk-1*	**20/**21.1	152/192 (2)	**21/**19.6	157/192 (2)	−4.8	0.8002
	*aak-2*	**23**/21.7	128/192 (2)	**21**/20.2	143/192 (2)	9.5	**0.0018**
	control	**26**/25.2	47/96(1)	**24**/21.6	63/96(1)	8.3	**0.0032**
	*daf-2*	**35**/34.9	74/96 (1)	**28**/26.4	67/96 (1)	25.0	**<0.0001**
	control	**23**/22.6	159/240 (2)	**21**/20.2	200/240 (2)	9.5	**<0.0001**
	*eat-2*	**21**/20.1	257/358 (2)	**23**/22.1	243/360 (2)	−8.7	**<0.0001**

a Median/mean RNAi lifespan of N2 and *hyl-1;lagr-1* fed the specified RNAi-bacteria.

b Some animals were censored as they crawled of the plate, ruptured, or died as a “bag of worms”, however they are incorporated in the data set up until the day they were censored. The number of individual trials is in parentheses.

c Median/mean control lifespan fed vector-only control bacteria.

d P-values were determined using the Gehan-Breslow-Wilcoxon test using GraphPad Prism version 6.0 (GraphPad Software). The Bonferroni method was used to correct for multiple comparisons and P- values below 0.0125 are considered statistically significant equivalent to a significance level of 0.05 with four pair-wise comparisons. Cumulative statistics is shown in this table as experimental animals subjected to the same treatment behaved similarly between trials. Data shown in Figure 1.

### Autophagy is Increased in *hyl-1;lagr-1* in a DAF-16 and SKN-1 Dependent Manner

**Figure 2:**
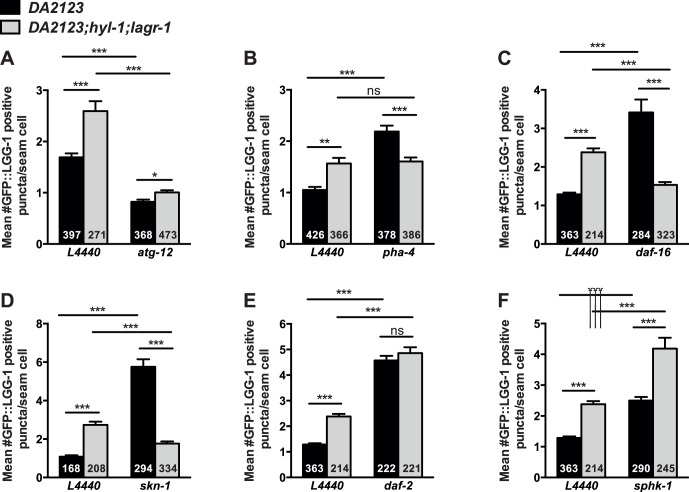
Autophagy is increased in *hyl-1;lagr-1* and the response mechanism differs from that of wild type. LGG-1 is part of autophagosomal membranes and widely used as an indicator of autophagy in *C. elegans*. Bars represent the mean number of LGG-1::GFP-containing puncta per seem cell in non-starved wild type and *hyl-1;lagr-1* worms grown at 20°C subjected to either empty vector control bacteria (L4440) or the indicated RNAi. The number in each bar indicates the total number of seam cells observed. (A) Knock-down of *atg-12* lowers the level of autophagy in both wild type and *hyl-1;lagr-1*. (B) Knock-down of *pha-4* does not change the increased level of autophagy in *hyl-1;lagr-1* but increases autophagy in wild type. (C) Knock-down of *daf-16* lowers the increased level of autophagy in *hyl-1;lagr-1* but increases autophagy in wild type. (D) Knock-down of *skn-1* lowers the increased level of autophagy in *hyl-1;lagr-1* but increases autophagy in wild type. (E) Knock-down of *daf-2* increases autophagy to the same extent in wild type and *hyl-1;lagr-1*. (F) Knock-down of *sphk-1* increases the level of autophagy in *hyl-1;lagr-1* beyond wild type level. Statistical analyses were performed by unpaired two-tailed t-test (with Welch’s correction if variances were significantly different) using GraphPad Prism version 6.0 (GraphPad Software). The Bonferroni method was used to correct for multiple comparisons and P values below 0.0125 were considered statistically significant equivalent to a significance level of 0.05. (*) P≤0.0125, (**) P≤0.001, and (***) P≤0.0001. N used for analysis is the total number of worms observed for each treatment (23–45 worms, two trials). Mean ± SEM is shown.

Since we identified ATG-12 function to be required for life span extension of *hyl-1;lagr-1* animals we examined the level of autophagy in these animals. Autophagy is commonly addressed by examining the formation of preautophagosomal structures using transgenic strains expressing the *C. elegans* LC3-homolog LGG-1 tagged with GFP, which associates to the autophagosomal membrane [11]. In order to visualize the level of autophagy in *hyl-1;lagr-1* animals, we introduced the *hyl-1(ok976)* and *lagr-1(gk331)* mutations into DA2123, a transgenic strain expressing GFP-tagged LGG-1. Under control conditions, *hyl-1;lagr-1* animals have a consistently increased level of autophagy compared to wild type worms (Figure 2A and Figure S4). 3-Methyladenine inhibits class III phosphatidylinositol-3-kinases and is commonly used as a specific inhibitor of formation of autophagosomes [20]. We observed that 3-MA prevented the increase in the number of autophagosomes in *hyl-1;lagr-1* animals (Figure S5). This and the observation that addition of concanamycin A (ConA), which blocks the lysosomal proton pump and thereby inhibits lysosome-dependent proteolysis [21], further increased the number of autophagosomes in both wild type and *hyl-1;lagr-1* animals (Figure S5), suggest that autophagy is induced in response to functional loss of HYL-1 and LAGR-1. Consistently, knock down of *atg-12* decreased the number of LGG-1::GFP positive puncta in both wild type and *hyl-1;lagr-1* animals (Figure 2A). Knock down of *pha-4* increased the number of LGG-1::GFP positive puncta in wild type worms, while *hyl-1;lagr-*1 animals were unresponsive to *pha-4* knock down (Figure 2B), indicating the autophagy response in *hyl-1;lagr-1* is independent of PHA-4. In contrast to this, we found that knock down of either *daf-16* or *skn-1* dramatically increased the level of autophagy in wild type, whereas *daf-16* or *skn-1* knock down in *hyl-1;lagr-1* animals reduced the number of LGG-1::GFP positive puncta to the level found in wild type fed control RNAi (Figure 2C and D), indicating the autophagic response in *hyl-1;lagr-1* depends on both DAF-16 and SKN-1. Despite that reduced IIS increases lifespan in both wild type and in *hyl-1;lagr-1* animals (Figure 1E), knock down of *daf-2* increased the level of autophagy to the same level in both wild type and in *hyl-1;lagr-1* animals (Figure 2E). These observations and the fact that increased autophagy in *daf-2* animals does not depend on DAF-16 [15] indicate that *hyl-1;lagr-1* is somehow more susceptible to lack of DAF-16. Both ceramide and sphingosine-1-phosphate have been shown to be able to induce autophagy [22]. While the induction of autophagy by sphingosine-1-phosphate during starvation is associated with a moderate induction promoting cell survival, induction of autophagy by ceramide is associated with a more comprehensive autophagy response leading to cell death [23]. We found that knock down of *sphk-1* increased the level of autophagy in both wild type and *hyl-1;lagr-1* to similar extents (Figure 2F), implying that increased levels of ceramide or a subset of ceramide species or reduced sphingosine-1-phosphate levels can induce autophagy. Since *sphk-1* knock down further increases the number of positive LGG-1::GFP puncta in *hyl-1;lagr-1*, induction of autophagy in *hyl-1;lagr-1* animals does not depend on sphingosine-1-phosphate *per se.* It is therefore interesting that knock down of *sphk-1* prevents the longevity extension of *hyl-1;lagr-1* animals, indicating that altered ceramide or sphingosine-1-phosphate levels can have diverse regulatory properties.

### *hyl-1;lagr-1* Animals Display Phenotypes Associated with Dietary-restricted Longevity

**Figure 3:**
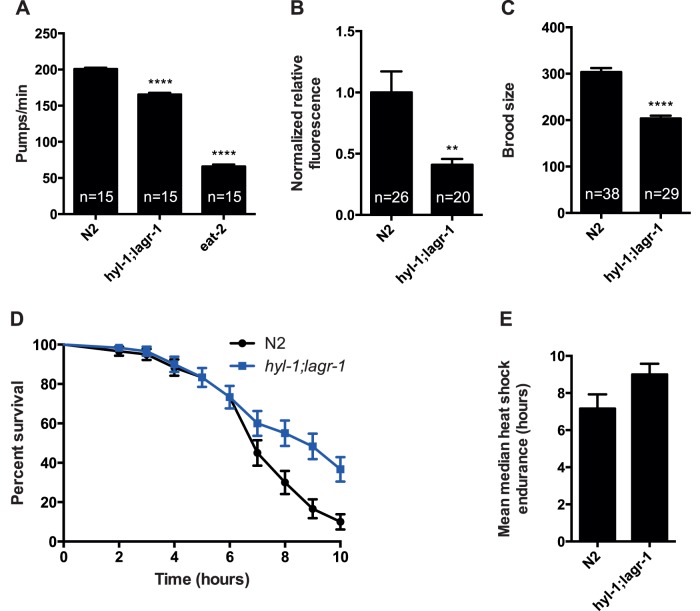
Phenotypes associated with lifespan extension and stress resistance are observed in *hyl-1;lagr-1*. (A) Pumping rates of N2, *hyl-1;lagr-1,* and an *eat-2* mutant. The latter displays a pronounced reduction in pumping rate and is commonly used as a genetic model for dietary restricted animals. Bars represent the mean number of pumps per minute. Compared to N2 displaying a mean pumping rate of 201±2 pumps/min, *hyl-1;lagr-1* shows a 17.4% decrease with a mean pumping rate of 166±2, P<0.0001, while *eat-2* shows a 67.2% decrease with a mean pumping rate of 66±3, P<0.0001. The data represents an mean ± SEM of 15 measurements in 5 worms of each genotype. (B) Quantification of fluorescent beads in the pharynx and the anterior part of the intestine following a feeding period of 30 minutes. Compared to N2, *hyl-1;lagr-1* displays 59% less fluorescence, P = 0.0026. Mean ± SEM is shown, n indicates the number of worms. (C) Mean total brood size of N2 and *hyl-1;lagr-1*. Compared to N2 which displays a mean brood size of 304±9, *hyl-1;lagr-1* shows a 33% decrease with a mean brood size of 204±6, P<0.0001. Mean ± SEM is shown, n = number of worms examined. (D) Survival curves of N2 and *hyl-1;lagr-1* subjected to heat-shock at 37°C. Compared to N2, *hyl-1;lagr-1* shows increased resistance, P = 0.0016. A total of 60 worms of each strain were assayed. Mean ± SD of 3 experiments is shown. N2-worms (6) and *hyl-1;lagr-1* worms (22) were censored but are incorporated in the analysis until the time they were censored. (E) Bars represent mean median heat shock survival from the 3 experiments shown in D. Compared to N2 which has a median survival of 7 hours, *hyl-1;lagr-1* displays a 29% increase in heat shock resistance with a median survival of 9 hours. Error bars represent ± SD.

The above described observations imply that *hyl-1;lagr-1* animals are dietary restricted. This prompted us to examine phenotypes, which are associated with dietary restriction. We found that *hyl-1;lagr-1* animals had a decreased pumping rate of 17.4% compared to N2 (Figure 3A, P<0.0001), whereas *eat-2* animals, which commonly are used as a genetic model for dietary restriction [17], displayed a 67.2% decrease in pumping rate compared to N2 (Figure 3A, P<0.0001). Consistently, we found that ingestion of bacteria mixed with fluorescent beads was reduced by 59% in *hyl-1;lagr-1* animals compared to control (Figure 3B, P = 0.0026), but was unchanged in *hyl-2* and in *hyl-2;lagr-1* animals (Figure S6). Moreover, we found that the brood size of *hyl-1;lagr-1* was 33% smaller than the brood size of N2 (Figure 3C, P<0.0001). Finally, increased thermotolerance has also been associated with dietary restriction [24] and consistently, we find that *hyl-1;lagr-1* showed increased resistance to heat-shock at 37°C (Figure 3D, *P* *= *0.0016 and Figure 3E,). Collectively, we interpret these observations as functional loss of HYL-1 and LAGR-1 renders *C. elegans* dietary restricted.

### Lipidomic Profiling Reveals Modified Sphingolipid Composition in *hyl-1;lagr-1*

**Figure 4:**
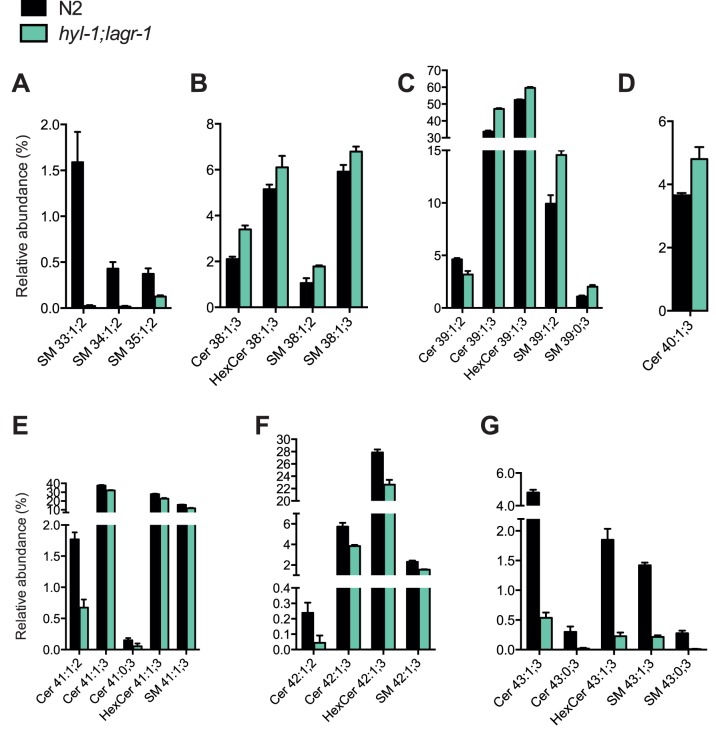
Lipidomic analysis reveals a modified sphingolipid composition in *hyl-1;lagr-1*. Relative abundance of detected sphingolipid species showing significant changes in *hyl-1;lagr-1*. Sphingolipids containing C24-26 fatty acids and sphingomyelin species containing C16-18 fatty acids are lowered in *hyl-1;lagr-1*, while sphingolipids containing C21-22 fatty acids acids are more abundant. *C. elegans* sphingolipids predominately contain C17 long-chain bases, thus making the fatty acid chain length readily deducible. The number of carbon atoms indicated is without head groups. (A) Sphingomyelins containing C16-18 fatty acids are significantly reduced in *hyl-1;lagr-1*. (B) All significantly changed sphingolipid species containing C21 fatty acids are more abundant in *hyl-1;lagr-1*. (C) All significantly changed sphingolipid species containing C22 fatty acids are more abundant in *hyl-1;lagr-1*. (D) The only significantly changed sphingolipid species containing C23 fatty acids is more abundant in *hyl-1;lagr-1*. (E) All significantly changed sphingolipid species containing C24 fatty acids are less abundant in *hyl-1;lagr-1*. (F) All significantly changed sphingolipid species containing C25 fatty acids are less abundant in *hyl-1;lagr-1*. (G) All significantly changed sphingolipid species containing C26 fatty acids are less abundant in *hyl-1;lagr-1*. Statistical analyses were performed by one way analysis of variance followed by Dunnett’s multiple comparisons test using GraphPad Prism version 6.0 (GraphPad Software). Results from two biological experiments are shown for each strain. Mean ± SD is shown. All species shown are at least significant different at the P<0.05 level. The entirety of detected sphingolipid species can be seen in Figure S7 and Figure S8.

To address the underlying molecular mechanisms governing dietary restriction of *hyl-1;lagr-1*animals, we used LC/MS to examine the global changes in ceramides, glycosylceramides, and sphingomyelin in each of the ceramide synthase mutants (Figure 4, Figure S7, and Figure S8). Consistent with previous observations [10], we found that the presence of HYL-1 is required for the formation of sphingomyelin (SM) species containing C16-18 and C26 fatty acid residues (e.g. SM33:1;2, SM35:1;2 and 43:1;3) as these were drastically reduced in *hyl-1* (Figure 4 and Figure S8C–D, J). We also found that HYL-2 has preference for incorporating C21-22 fatty acids (e.g. Cer/HexCer/SM 39:1;3) (Figure S8E–F) while HYL-1 appears to have a higher affinity towards incorporating C26 fatty acids primarily (e.g. Cer/HexCer/SM 43:1;3) (Figure S8J). The abundance of sphingolipids containing C22 fatty acids (e.g. Cer39:1;3 and HexCer39:1;3) was increased in *hyl-1;lagr-1* animals (Figure 4C), while the levels of sphingolipids containing C24-26 fatty acids (Figure 4E–G) as well as sphingomyelin species containing C16-18 fatty acid moieties (SM 33–35:1;2) (Figure 4A) were much lower compared to wild type animals.

The fact that loss of function of the different ceramide synthases differentially affect lifespan of *C. elegans*, prompted us to identify unique changes in the sphingolipidomes of each of these strains, which correlated with the observed lifespans. We found that the total level of sphingomyelin species containing C21-22 fatty acids (e.g. SM38:1;3 and SM39:1;3) was lower in *hyl-2* and *hyl-2;lagr-1* animals while increased in *hyl-1;lagr-1*(Figure S7C, Figure S8E–F). However, two sphingomyelin species containing C24 and C25 fatty acids (SM 41/42:1;3) was increased in *hyl-2* and *hyl-2;lagr-1* and lowered in *hyl-1;lagr-1* (Figure S7C, Figure S8H–I). Interestingly, deletion of *lagr-1* only had subtle effects on the ceramide and sphingolipid composition and level (Figure S8D–J). However, loss of LAGR-1 function in *hyl-2* animals prevented sphingomyelins containing C16-C18 fatty acid moieties (SM 33–35:1;2) to increase as they do in *hyl-2* animals (Figure S8C–D), arguing that LAGR-1 primarily contributes to the synthesis of long-chain fatty acid containing sphingomyelins.

These observations indicate that the SM species increased in *hyl-1;lagr-1* and lowered in *hyl-2* and *hyl-2;lagr-1* may have a pro-longevity function while the SM species with the opposite distribution have pro-aging effects. These species can be seen in Figure S8K. Thus, we performed Principal Component Analysis to further address this model. The score plot of the sphingolipidomics data clustering showed a clear distinction between the three groups; “Decreased lifespan (*mutant*)”, “Increased lifespan (*mutant*)”, and “No change (ctrl N2)” (Figure S9). The corresponding loading plot revealed that Cer 41:1;3, HexCer 39:1;3, HexCer 41:1;3, SM 38:1;2, SM 39:0;2, SM 39:1;2, SM 41:1;3, SM 41:1;3 contributed the most to the score plot separation (Figure S9A–B). Among these species only the abundance of SM 38:1;2 and partly SM 39:1;2 and SM 41:1;3 (Figure S9C–E) are oppositely regulated taking all three longevity phenotypes into account, suggesting that the levels of these sphingolipid species may play a role in regulating longevity of *C. elegans*. We detected additional sphingolipid species differing between *hyl-1* animals and *hyl-1;lagr-1* animals (Figure S9B, D–E, Table S1). These observations may not directly account for the extended longevity of *hyl-1;lagr-1* animals, however, it may indicate that specific sphingolipid species are involved in the longevity response. It also supports the notion that specific species within sphingolipid subgroups may have distinct functions and that these subgroups of lipid species cannot necessarily be viewed as units having the same properties and acting in the same pathways.

## Discussion

The important role of ceramide synthases in organismal aging was first recognized when it was found that loss of the gene encoding the ceramide synthase Lag1 in the yeast *Saccharomyces cerevisiae* extended chronological lifespan [25], which is consistent with the recent observations that both genetic- and chemical inhibition of sphingolipid synthesis extend chronological lifespan of yeast [26]. Moreover, it has been shown that homologues of *LAG1* from *C. elegans* not only complement Lag1 function in *S. cerevisiae* [7], [27], [28] but also differentially are required for normal longevity in *C. elegans* [8], [10]. In the present study we have further examined the role of ceramide synthases in aging in *C. elegans* and show that deletion of *hyl-1*(*ok976*) or *lagr-1*(*gk331*) does not affect lifespan (Figure S2), while deletion of *hyl-2*(*ok1766*) significantly shortens lifespan of *C. elegans*. Interestingly, we consistently find that deletion of both *lagr-1* and *hyl-2* further reduces lifespan, while deletion of both *lagr-1* and *hyl-1* significantly extends longevity compared to wild type *C. elegans* (Figure 1 and Figure S2). We did not succeed in generating *hyl-1;hyl-2* animals, which is consistent with the observation that knock down of *hyl-1* in *hyl-2*(*ok1766*) animals results in L1 arrest (data not shown). In contrast to our observations, Menuz et al. [10] found that loss of HYL-1 function (*ok976*) extends lifespan significantly, while deletion of *hyl-2*(*ok1766*) did not affect lifespan under normal conditions, but was required for survival under anoxic conditions [10]. Thus, our finding suggests that HYL-2 is not only required for survival at low oxygen tension, but also required for normal lifespan. We find that the extended lifespan of *hyl-1;lagr-1* animals is independent of insulin signaling (Figure 1), but depends on ATG-12, a component of the autophagic machinery [29], and the three central transcription factors DAF-16, SKN-1, and partly PHA-4 (Figure 1), which previously have been shown to modulate aging under dietary restriction [14], [30]. Thus, loss of HYL-1 and LAGR-1 function may therefore induce a dietary restriction-like phenotype, which ultimately leads to extension of longevity. This scenario is supported by our observation that knock down of *eat-2* does not further extend lifespan of *hyl-1;lagr-1* animals (Figure 1), and by data showing that *hyl-1;lagr-1* animals have decreased pumping rate, ingestion, and brood size, and increased resistance towards heat stress (Figure 3), which are all hallmarks of dietary restriction [31]. Furthermore, one of the best evolutionarily conserved cellular responses to dietary restriction is the activation of autophagy, a lysosomal degradation pathway in which the cell self-digests its own components to provide nutrients to maintain crucial cellular functions during fasting. Accordingly, the number of autophagosomes is significantly increased in *hyl-1;lagr-1* animals. Interestingly, we find that knock down of *atg-12*, *skn-1,* and *daf-16* in *hyl-1;lagr-1* animals diminishes the number of GFP::LGG-1 positive puncti to wild type levels, while knock down of *pha-4* does not increase the number of autophagosomes further as we observe in control animals (Figure 2). We also find that knock down of *daf-2* expression increases the number of LGG-1::GFP positive puncti in both control and in *hyl-1;lagr-1* animals. Thus, our results indicate that both lifespan extension and the increased number of autophagosomes in *hyl-1;lagr-1* animals depends on a functional autophagy machinery as well as the transcription factors DAF-16, SKN-1, and partly PHA-4, and is independent of the insulin signaling pathway (Figure 2).

Our findings suggest that the lifespan extension of *hyl-1;lagr-1* animals is not only mediated by a few genes, but rather a complex network of gene functions. This is in agreement with previous findings showing that the lifespan extension of *eat-2* mutants, which commonly is used as a genetic surrogate for dietary restriction, depends on PHA-4 function [13], [14], while SKN-1 and PHA-4 both are required for lifespan extension by dietary restriction in liquid media [13], [16]. In contrast to these observations, the lifespan extension induced by dietary restriction on solid media depends on AAK-2 and DAF-16 [32].

Consistent with previous findings our lipidomic profiling supports the notion that HYL-1 promotes synthesis of ceramides containing very-long acyl-chains, while HYL-2 confers synthesis of ceramide species with shorter fatty acid moieties (Figure S8) [10]. Notably, we find that deletion of *lagr-1* has only subtle effects on the level and molecular composition of sphingolipids, independent of the presence of functional *hyl-1* and *hyl-2* (Figure S8A–K). Despite its minor contribution to the overall molecular sphingolipid species composition, LAGR-1 contributes to *C. elegans* longevity as *hyl-1;lagr-1* animals live significantly longer while *hyl-1* and *lagr-1* animals do not (Figure S2). This and the fact that HYL-1, HYL-2, and LAGR-1 are expressed in different tissues (Figure S10), suggest that impaired synthesis of specific ceramide species in a limited number of cells can induce a DR-like response. Our observation that ingestion is severely reduced in *hyl-1;lagr-1* animals (Figure 3) and the fact that impaired synthesis of glycosphingolipids by ceramide glucosyltransferases in a subset of cells in the digestive tract impairs feeding [9] lend credence to the notion that loss of specific ceramides and sphingolipids are required for specific cellular functions in a subset of tissues. Principal component analyses of the sphingolipidomic data (Figure S9) indicate that the abundance of specific sphingolipids like SM 38:1;2, 39:1;2, 39:1;3, 41:1;3, and HexCer 39:1;3 could contribute to the diminished lifespan of *hyl-2* animals, while the level of SM 38:1;2 could contribute to the lifespan extension of *hyl-1;lagr-1* animals.

Our results show that primarily HYL-1 and HYL-2 functions contribute to the overall abundance and molecular composition of ceramide and other sphingolipids in *C. elegans*. Despite the subtle contribution of LAGR-1 to the synthesis of these lipids, deletion of *lagr-1* further impairs longevity of *hyl-2* animals, while deletion of *lagr-1* extends the lifespan of *hyl-1* animals. The observation that knock down of *sphk-1* expression decreases *hyl-1;lagr-1* lifespan to wild type level but increases the number of autophagosomes in *hyl-1;lagr-1* animals, suggests that the longevity response to *sphk-1* knock down is induced by mechanisms, which do not involve autophagy. Interestingly, although our observations supports the notion that autophagy supports cell survival, recent studies have shown that autophagy can promote cell death under certain conditions (reviewed in [33]). Accordingly, it has recently been found that chemical inhibition of sphingosine kinase 2 induces the formation of autophagosomes while promoting nonapoptotic cell death in human kidney carcinoma cells [34].

While the present work shows that synthesis and turn-over of sphingolipids and sphingosine-1-phosphate(s) can regulate the longevity and autophagy, the genetic tractability of *C. elegans* and the mutant strains we have obtained so far provide an excellent framework to further delineate how specific ceramide and sphingolipid species regulate organismal longevity and autophagy.

## Materials and Methods

### Strains

Standard procedures were used for culturing [35]. Single mutants: RB1036: *hyl-1(ok976) IV*, RB1498: *hyl-2(ok1766) X*, VC765: *lagr-1(gk331) I*, DA1116: *eat-2(ad1116) II*. Double mutants: FE0007: *hyl-1(ok976) IV;lagr-1(gk331) I*, FE0008: *hyl-2(ok1766) X; lagr-1 (gk331) I*. Transgenic strains: DA2123: adIS2122[*lgg-1::lgg-1-GFP; rol-6(su1006)*] (kind gift from Malene Hansen, Sanford-Burnham Medical Research Institute, La Jolla, California), FE0015: *hyl-1(ok976) IV;lagr-1(gk331) I*; adIS2122[*lgg-1::lgg-1-GFP; rol-6(su1006)*], BC10421: *dpy-5(e907)* I; sEx10421[*rCesC09G4.1::GFP+pCeh361*], VB2360: svEX798-800 [*hyl-2::GFP;rol-6 (su1006)*], BC10482: *dpy-5(e907)* I; sEX10482[*lagr-1::GFP*]. All single mutant strains were obtained from the *Caenorhabditis* Genetics Centre (CGC) and unless otherwise noted generation of the remaining strains listed were conducted in our lab. Generating a *hyl-1;hyl-2* strain was also attempted but worms homozygous for both mutations were not attained and we later confirmed by RNAi that worms lacking both HYL-1 and HYL-2 arrest development and die. The three single mutant strains were out-crossed seven times to N2 Bristol wild type worms.

### Confocal Microscopy

Transgenic L4 worms expressing GFP fusions were mounted in M9 buffer containing Tetramisol (10 µM) (Sigma-Aldrich, St. Louis, Montana, USA) on a 2% agar pad on a microscope slide and covered with a coverslip and analyzed by confocal microscopy on an LSM 510 META microscope (Carl Zeiss MicroImaging Inc., Germany). Primary image analysis was performed using LSM Image Browser (Carl Zeiss MicroImaging Inc., Germany).

### RNAi

All clones were from the Julie Ahringer RNAi library [36]. RNAi by feeding was performed according to Ahringer [37].

### Lifespan Analysis

All lifespan analyses were conducted at 20°C on worms non-starved for at least two generations. For the RNAi lifespan assays, 3-day-old synchronized worms were transferred to gene-specific RNAi bacterial plates. The worms were counted and moved to new plates every second day during the reproductive period and afterwards only moved to new plates every forth day. Lifespan is defined as the time from day one of adulthood to the time when they were scored as dead (i.e. no longer responded to gentle prodding with a platinum wire). Worms that “exploded”, were bagged, or went missing were censored the day the event was observed. For the OP50 lifespan assays (Figure S2), synchronized L4 worms were transferred to plates containing 100 µM FUDR (5-fluoro-2′-deoxyuridine, 50503 Sigma) to prevent progeny from developing and counted every 1–2 days. Lifespan is defined as the time from when the worms were placed on FUDR plates to the time when they were scored as dead.

### Autophagy Assay

The level of autophagy was investigated using an LGG-1::GFP translational reporter [11]. GFP positive puncta/seem cell were counted in L4 transgenic worms at 1000× magnification using a Leica DMI 6000 B microscope. Counting puncta is usually performed in L3 animals as autofluorescence increases with age and obscures LGG-1::GFP visualization. We investigated the level of autophagy in young L4 worms in order to age-match the assay with the feeding and pumping rate assays. All animals were kept at 20°C and assayed by the same experimenter. The number of autophagosomes per seem cell was averaged for each worm and this average was used for calculating the mean number of LGG-1::GFP containing puncta/seem cell.

### Feeding Assay

NGM plates were seeded with a 500∶1(vol:vol) blend of OP50 bacteria and Fluoresbrites Multifluorescent microspheres, 0.2 µm (Polyscience, Inc.) 22 hours prior to the assay. L4 larvae of N2 and *hyl-1;lagr-1* were allowed to feed for 30 min on the bead-plates after which they were transferred to eppendorf tubes with 1 ml of 10 mM levamisole (Sigma-Aldrich, St. Louis, Montana, USA) and centrifuged for 2 min at 2000 g, room temperature. The supernatant was removed and ∼30 µl of resuspended worms were transferred to a freshly made agarose pad (2% agarose in M9). The amounts of ingested beads were photographed at 1000× using a Leica DMI 6000 B microscope and quantified using NIH ImageJ freeware [38]. All images were acquired using identical settings and exposure time and the presented images, are representative of general observations.

### Pumping Rate Assay

Five non-starved L4 worms of each strain were transferred to NGM plates seeded with OP50 and filmed three times 15 consecutive seconds using a Leica DMI 6000 B microscope with Infinity Capture Software. Pumping rates were counted by playing the recordings at one fourth of the normal speed and mean pumping rate calculated from the fifteen measurements of each genotype.

### Brood Size Assay

Brood size assays were performed at 20°C by picking late L4s to fresh NGM plates and transferring them to new plates every 24 hours for 4–5 days. After this worms were left on the same plates for 3 days. Worms that “exploded”, were bagged, or went missing were omitted from the analysis. All progeny plates were incubated at 20°C for about two days before the number of developed progeny was scored.

### Heat Shock Assay

Synchronized L4 worms grown at 20°C were picked to fresh plates and subjected to heat shock at 35°C for one hour after which the worms were counted. Worms were scored dead when they no longer responded to gentle prodding with a platinum wire. The worms were continuously subjected to heat stress one hour followed by scoring.

### RNA Isolation, cDNA Synthesis, and Quantitative Real-time PCR

Total RNA was extracted from four independent synchronized worm populations of each strain as described (51). cDNA was synthesized from 500 ng total RNA as previously described (52). Quantitative real-time PCR (qRT-PCR) was performed on an ABI PRISM 7700 RealTime PCR-machine (Applied Biosystems, Carlsbad, California, USA) or Stratagene MXPro 3000 (Agilent Technologies, Santa Clara, California, USA) using 2× SYBR Green JumpStart™ Taq ReadyMix™ and Sigma Reference Dye (Sigma-Aldrich, St. Louis, Montana, USA) as described by the manufacturer. PCR reactions were performed in 25 µl reactions containing 1.5 µl diluted cDNA. Reactions were incubated at 95°C for 2 min followed by 40 cycles of 95°C for 15 s, 60°C for 45 s and 72°C for 45 s. All reactions were performed in duplicates and normalized to the level of the *tbb-2* gene (encoding the *C. elegans* orthologue of the human β-tubulin). Primers for qRT-PCR were designed using Primer Express version 2.0 (Applied Biosystems, Carlsbad, California, USA). Primer sequences can be obtained upon request. Statistical analyses were performed by unpaired two-tailed t-test using GraphPad Prism version 6.0 (GraphPad Software).

### Lipid Extraction

Synchronized L4 worms were washed twice with 10 mL 0.9% NaCl and incubated end-over-end for 15 min to ensure intestinal emptying. Worms were washed trice with 10 mL 150 mM ammonium acetate in H_2_O. All centrifugation steps were carried out at 20°C, 1000 rpm, 1 min. Liquid was aspirated to 600 µL and 550 µl were transferred to an eppendorf tube. Samples were flash frozen in liquid nitrogen and stored at −80°C. Lipid extraction was carried out at 4°C. Approximately 1000 worms were aliquotted (∼100 µl) mixed with 100 µl 150 mM ammonium acetate before adding 990 µl chloroform/methanol (2∶1). Samples were vortexed at 4°C for 45 min and centrifuged 2 min (2000 **g**, 4°C). The lower organic phase was transferred to a new tube and subjected to vacuum evaporation. Lipid extracts were dissolved in 100 µl chloroform/methanol (2∶1).

### Lipid Analysis by Mass Spectrometry

Lipid extracts were analyzed by normal-phase liquid chromatography using a PVA-SIL column (YMC Europe GmbH) interfaced with a nanoflow ion source Triversa NanoMate (Advion Biosciences, Inc.) and a LTQ Orbitrap XL mass spectrometer (Thermo Fisher Scientific). Cer, HexCer and SM species were monitored in negative ion mode by recording FT MS analysis using a target mass resolution of 100,000. Lipid species were quantified by extracting their peak intensities and using ALEX software as previously described [39]. The relative abundance of lipid species was determined by normalizing the intensity of monitored sphingolipid species to the total intensity of all monitored lipid species. Sphingolipid species was annotated according to previous reports [39]–[41].

### Statistical Analyses

All statistical analyses were performed using GraphPad Prism version 6.0 (GraphPad Software), except for the Principal Component Analysis (PCA), which was performed using Markerview software (AB SCIEX).

#### Lifespan analysis

Statistical analyses were performed by Gehan-Breslow-Wilcoxon tests. The Bonferroni method was used to correct for multiple comparisons and P-values equivalent to a significance level of 0.05 were considered statistically significant. Worms that “exploded”, were bagged, or went missing were censored the day the event was observed. Further statistical lifespan details can be found in Table 1 and Figure S2.

#### Autophagy assay

Statistical analysis was performed by unpaired two-tailed t-test (with Welch’s correction if variances were significantly different). The Bonferroni method was used to correct for multiple comparisons and P-values equivalent to a significance level of 0.05 were considered statistically significant. N used for analysis is the total number of worms observed for each treatment.

#### Feeding assay and quantitative real-time PCR

Statistical analyses were performed by unpaired two-tailed t-test (with Welch’s correction if variances were significantly different).

#### Pumping rate and brood size assays

Statistical analyses were performed by one way analysis of variance followed by Dunnett’s multiple comparisons test.

#### Heat shock assay

Statistical analysis was performed by Log-rank (Mantel-Cox) tests. Some animals were censored as they crawled of the plate or ruptured, however they are incorporated in the data set up until the time point they were censored.

#### Lipid analysis by mass spectrometry

Statistical analyses were performed by one way analysis of variance followed by Dunnett’s multiple comparisons test and P-values below 0.05 were considered statistically significant. Results are from two biological experiments.

#### Principal component analysis

Supervised principal component analysis was performed with the following preprocessing parameters; Weighting = none, Scaling = Pareto. We excluded species which were not significantly altered between at least two of the three lifespan categories: “No change”, “Increased lifespan”, and “Decreased Lifespan”. P-values from the t-test of *hyl-1* and *hyl-1;lagr-1* (Table S1) have been corrected for multiple comparisons and accordingly, P-values below 0.05 are considered statistically significant.

## Supporting Information

Figure S1 **Structure examples of selected sphingosine-, ceramide-, sphingomyelin- and glucosyl ceramide species from** ***C. elegans*** **.**
(PDF)

Figure S2 **Lifespan analyses of all ceramide synthase mutants.** Cumulative lifespan analyses of N2, *hyl-1, hyl-2, lagr-1, hyl-1;lagr-1,* and *hyl-2;lagr-1* grown on OP50 at 20°C. (A) *hyl-1* displays no change in lifespan compared to N2 (P = 0.3592). (B) *hyl-2* displays a shortened lifespan compared to N2 (P<0.0001). (C) *lagr-1* displays no change in lifespan compared to N2 (*P* = 0.1651). (D) *hyl-1;lagr-1* displays an extended lifespan compared to N2 (P<0.0001). (E) *hyl-2;lagr-1* displays shortened lifespan compared to N2 (P<0.0001). (F) Table summarizing the depicted lifespan analyses. ^a^Some animals were censored as they crawled of the plate, ruptured, or died as a “bag of worms”, however they are incorporated in the data set up until the day they were censored. The number of individual trials is in parentheses. Statistical analyses were performed by Gehan-Breslow-Wilcoxon tests using GraphPad Prism version 6.0 (GraphPad Software). ^b^The Bonferroni method was used to correct for multiple comparisons and P-values below 0.01 are considered statistically significant equivalent to a significance level of 0.05. Cumulative statistics is shown in this table as experimental animals subjected to the same treatment behaved similarly between trials.
(PDF)

Figure S3 **Expression levels of longevity and autophagy genes in wild type N2 and in** ***hyl-1;lagr-1*** **animals.** Total RNA was harvested from N2 worms at the L4 stage. The expression level of the indicated genes was quantified using qRT-PCR, and normalized to *tbb-2* mRNA and shown in arbitrary units (a. u.). Mean ± SEM is shown, number of independent experiments ranged from 3 to 9. (*) P≤0.05. Only the expression of *pha-4* and *atg-12* in *hyl-1;lagr-1* animals is significantly changed relatively to wild type animals.
(PDF)

Figure S4 **LGG-1::GFP-positive puncta in hypodermal seam cells of wild type and** ***hyl-1;lagr-1*** **animals.** Representative micrographs of young L4 N2 (DA2123) or *hyl-1;lagr-1* larvae expressing GFP-tagged LGG-1 in hypodermal seam cells. A yellow arrow head indicates a hypodermal seam cell, while blue arrow heads indicate LGG-1::GFP positive puncti. Using fluorescence microscopy, LGG-1::GFP positive puncti were counted in 3–10 seam cells were counted in each of 23–45 animals and averaged. Scale bar: 20 µm.
(PDF)

Figure S5 **Effect of 3-methyladenine and Concanamycin A on autophagy**. Transgenic animals expressing LGG-1::GFP were treated with 3-MA (1 mM) or Concanamycin A (50 nM) for 24 hours after they reaching the young L4 larval stage. Bars represent mean number of LGG-1::GFP-containing puncta per seem cell in non-starved wild type and *hyl-1;lagr-1* worms grown at 20°C. The number in each bar indicates the total number of seam cells observed. N used for analysis is the total number of worms observed for each treatment (the number of worms examined ranged from 12 to 21). Mean ± SEM is shown. (**) P≤0.01 and (****) P≤0.0001.
(PDF)

Figure S6 **Ingestion of fluorescent beads in** ***hyl-2*** **and in** ***hyl-2;lagr-1*** **animals.** Quantification of fluorescent beads in the pharynx and the anterior part of the intestine following a feeding period of 30 minutes. Fluorescence intensities were normalized to the level in wild type animals. Mean ± SEM is shown, n = number of worms analyzed.
(PDF)

Figure S7 **Overview of all Cer, HexCer, and SM species detected in** ***hyl-1;lagr-1*** **.** (A) Top: Relative abundance of all Cer species detected in *hyl-1;lagr-1*. Bottom: Zoom of low abundance Cer species detected in *hyl-1;lagr-1*. (B) Relative levels of all HexCer species detected in *hyl-1;lagr-1*. (C) Top: Relative abundance of all SM species detected in *hyl-1;lagr-1*. Bottom: Zoom of low abundance SM species detected in *hyl-1;lagr-1*.
(PDF)

Figure S8 **Overview of all Cer, HexCer, and SM species detected in the five different ceramide synthase mutants.** (A) Top: abundance levels of all Cer species detected in ceramide synthase mutants. Bottom: Zoom of low abundance Cer species detected in ceramide synthase mutants. (B) Top: Relative levels of all HexCer species detected in ceramide synthase mutants. Bottom: Zoom of low abundance HexCer species detected in ceramide synthase mutants. (C) Top: Relative levels of all SM species detected in ceramide synthase mutants. Bottom: Zoom of low abundance SM species detected in ceramide synthase mutants. (D) Relative levels of all C33, C34, and C35 sphingolipid species detected to have significantly different levels in one or more ceramide synthase mutant compared to N2. (E) Relative levels of all C38 sphingolipid species detected to have significantly different levels in one or more ceramide synthase mutant compared to N2. (F) Relative levels of all C39 sphingolipid species detected to have significantly different levels in one or more ceramide synthase mutant compared to N2. (G) Relative abundance of all C40 sphingolipid species detected to have significantly different levels in one or more ceramide synthase mutant compared to N2. (H) Relative levels of all C41 sphingolipid species detected to have significantly different levels in one or more ceramide synthase mutant compared to N2. (I) Relative levels of all C42 sphingolipid species detected to have significantly different levels in one or more ceramide synthase mutant compared to N2. (J) Relative levels of all C43 sphingolipid species detected to have significantly different levels in one or more ceramide synthase mutant compared to N2. (K) Relative levels of all species significantly altered according to lifespan changes (e.g. Oppositely regulated in long-lived and short-lived strains). Statistical analyses were performed by one way analysis of variance followed by Dunnett’s multiple comparisons test using GraphPad Prism version 6.0 (GraphPad Software). Two technical replicates of two biological replicates were analysed for each strain. (*) P≤0.05, (**) P≤0.001, and (***) P≤0.0001. Mean ± SD is shown.
(PDF)

Figure S9 **Multivariate analysis by principal component analysis of sphingolipidomic data segregated according to lifespan changes.** (A) Score plot projecting the first (D1) and second (D2) principal components show a clear separation of the strains when grouped according to the following lifespan changes: “Decreased lifespan ”, “Increased lifespan ”, and “No change ”. D1 and D2 account for 100% of the total sample variance. **(B)** Loading plot depicting the sphingolipid species contributing the most to the total sample variance. Red stars denotes the species significantly altered when comparing *hyl-1* (no lifespan change) to *hyl-1;lagr-1*(increased lifespan). **(C)** Relative abundance of the four species contributing the most to the score plot separation. **(D and E)** The 5 species which contribute most to the separation and are significantly altered in *hyl-1;lagr-1* compared to *hyl-1* (denoted by red stars in B).
(PDF)

Figure S10 **Expression patterns of the ceramide synthase genes at the L4 stage.** (A) HYL-1 shows expression in the body wall muscles, the pharyngeal muscles PM3 and PM5, and unidentified cells in the pharynx. (B) HYL-2 shows expression in the body wall muscles and the nervous system. (C) LAGR-1 shows expression in the pharyngeal muscles PM3-5 and in pharyngeal nerves. Scale bar: 80 µm.
(PDF)

Table S1 **P-values from the t-test of** ***hyl-1*** **and** ***hyl-1;lagr-1*** **of the Principal Component Analysis.**
(XLSX)
