# Supplementary figures and images for: Functional Loss of Two Ceramide Synthases Elicits Autophagy-Dependent Lifespan Extension in C. elegans
Source: PLoS One. 2013 Jul 19;8(7):e70087. doi: 10.1371/journal.pone.0070087 (PMC3716707; doi:10.1371/journal.pone.0070087)

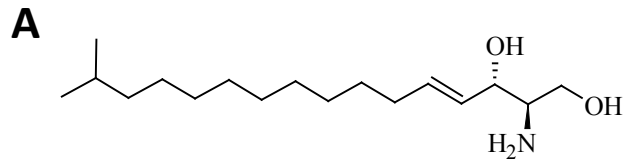

**Sphingosine** (d17:1;2)

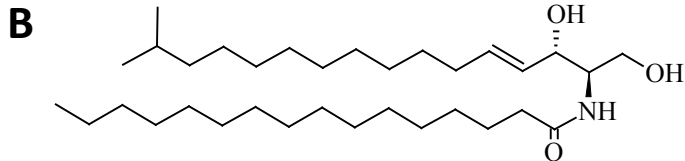

**Ceramide 33:1;2** (d17:1;2/16:0:0)

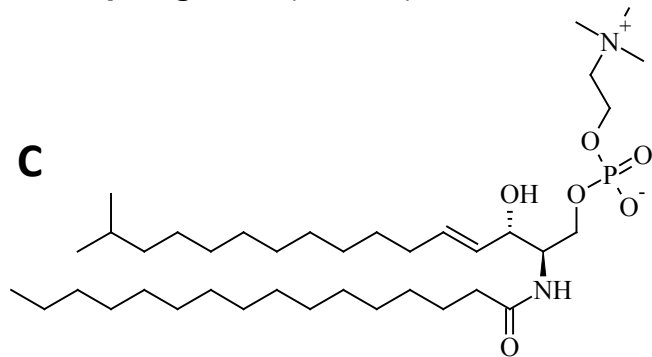

**SM 33:1;2** (d17:1;2/16:0:0)

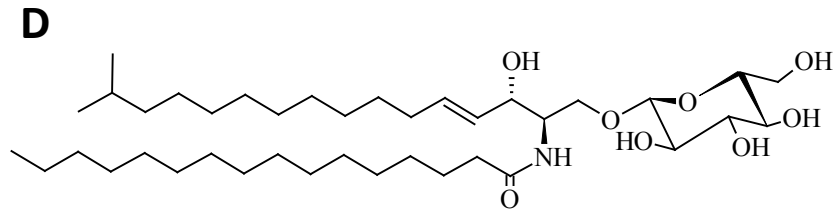

**Glucosyl Ceramide 33:1;2** (d17:1;2/16:0:0)

Supplement: Figure S1 — Structure examples of selected sphingosine-, ceramide-, sphingomyelin- and glucosyl ceramide species from C. elegans . (PDF) [file pone.0070087.s001.pdf]

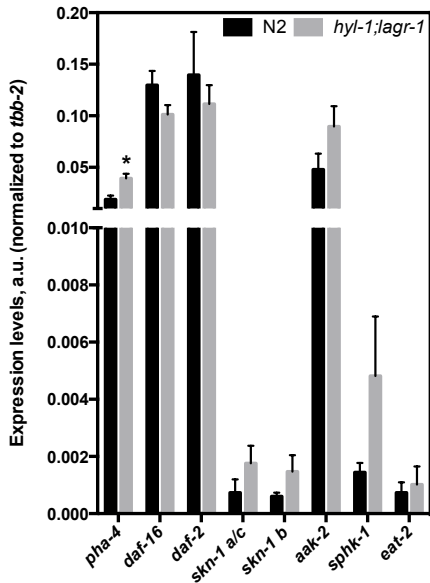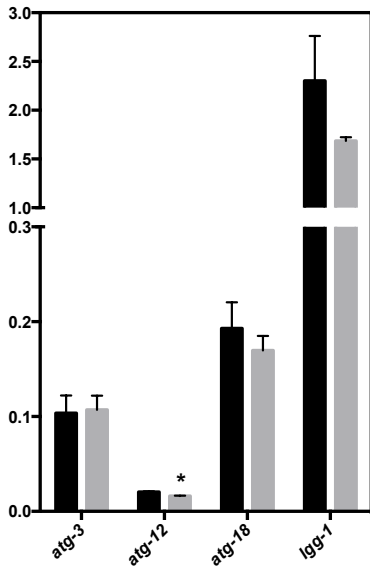

Figure S3

Supplement: Figure S3 — Expression levels of longevity and autophagy genes in wild type N2 and in hyl-1;lagr-1 animals. Total RNA was harvested from N2 worms at the L4 stage. The expression level of the indicated genes was quantified using qRT-PCR, and normalized to tbb-2 mRNA and shown in arbitrary units (a. u.). Mean ± SEM is shown, number of independent experiments ranged from 3 to 9. (*) P≤0.05. Only the expression of pha-4 and atg-12 in hyl-1;lagr-1 animals is significantly changed relatively to wild type animals. (PDF) [file pone.0070087.s003.pdf]

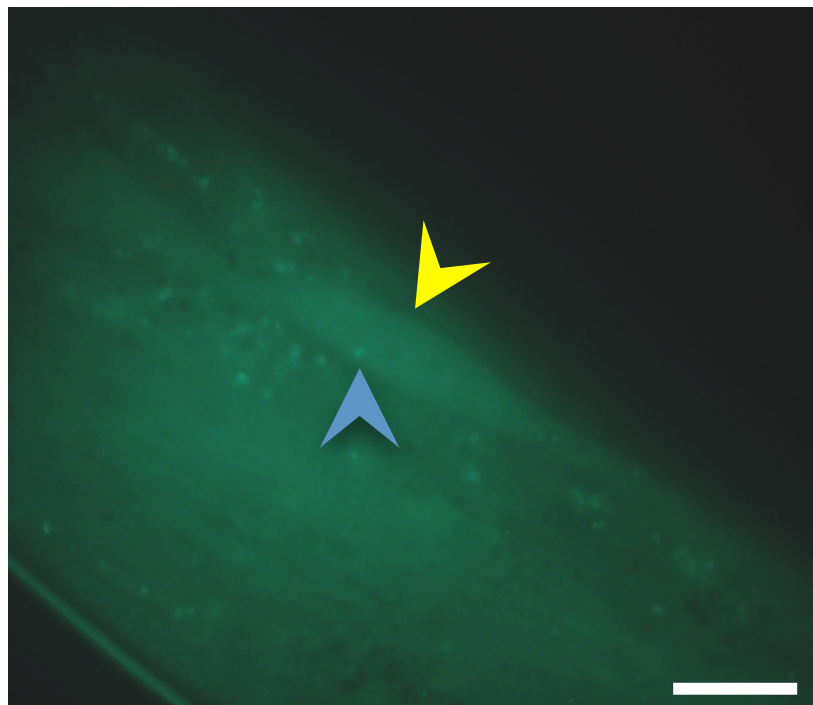

*DA2123*

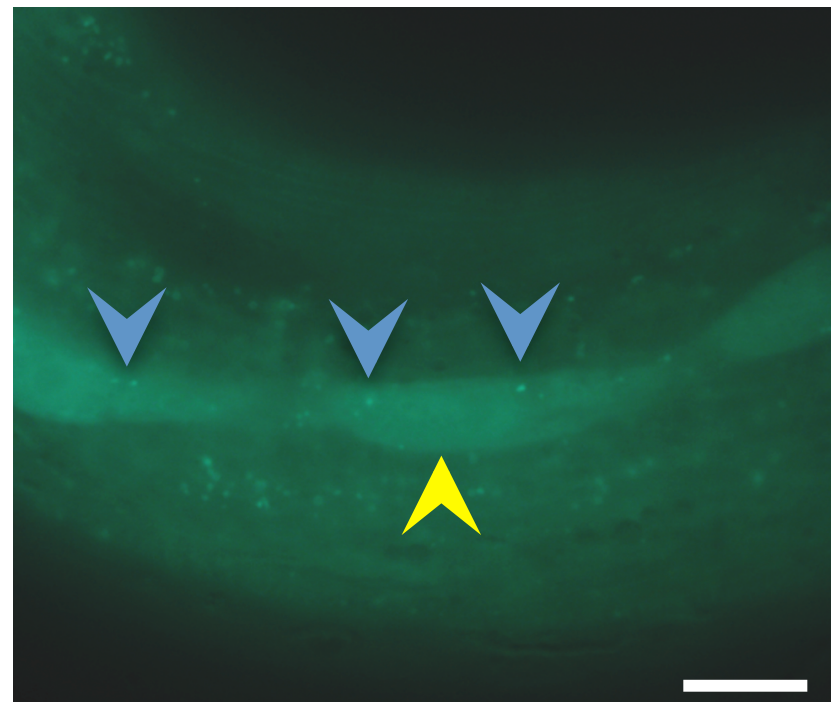

*DA2123;hyl-1;lagr-1*

Supplement: Figure S4 — LGG-1::GFP-positive puncta in hypodermal seam cells of wild type and hyl-1;lagr-1 animals. Representative micrographs of young L4 N2 (DA2123) or hyl-1;lagr-1 larvae expressing GFP-tagged LGG-1 in hypodermal seam cells. A yellow arrow head indicates a hypodermal seam cell, while blue arrow heads indicate LGG-1::GFP positive puncti. Using fluorescence microscopy, LGG-1::GFP positive puncti were counted in 3–10 seam cells were counted in each of 23–45 animals and averaged. Scale bar: 20 µm. (PDF) [file pone.0070087.s004.pdf]

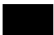 DA2123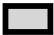 DA2123;*hyl-1*;*lagr-1*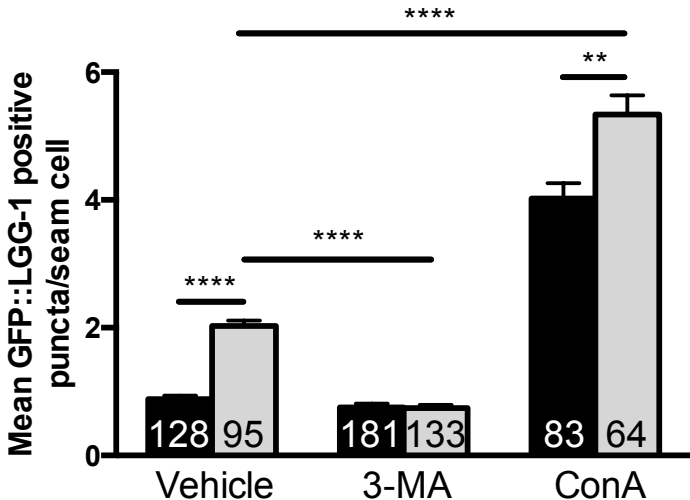

Supplement: Figure S5 — Effect of 3-methyladenine and Concanamycin A on autophagy. Transgenic animals expressing LGG-1::GFP were treated with 3-MA (1 mM) or Concanamycin A (50 nM) for 24 hours after they reaching the young L4 larval stage. Bars represent mean number of LGG-1::GFP-containing puncta per seem cell in non-starved wild type and hyl-1;lagr-1 worms grown at 20°C. The number in each bar indicates the total number of seam cells observed. N used for analysis is the total number of worms observed for each treatment (the number of worms examined ranged from 12 to 21). Mean ± SEM is shown. (**) P≤0.01 and (****) P≤0.0001. (PDF) [file pone.0070087.s005.pdf]

Figure S6

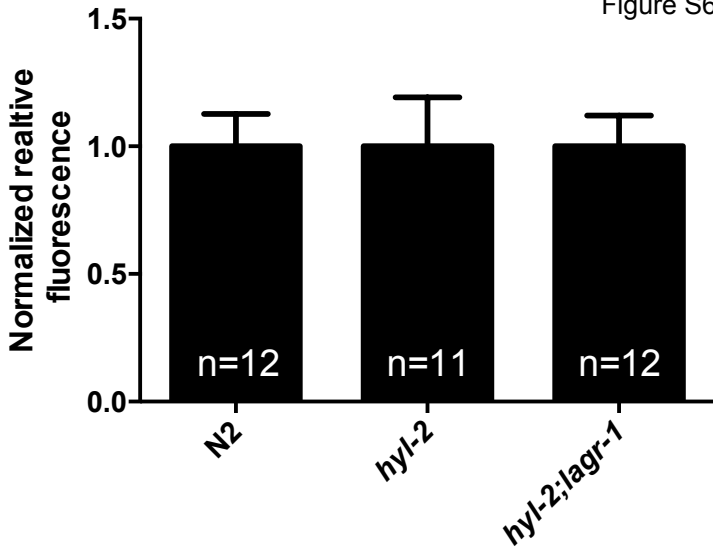

Supplement: Figure S6 — Ingestion of fluorescent beads in hyl-2 and in hyl-2;lagr-1 animals. Quantification of fluorescent beads in the pharynx and the anterior part of the intestine following a feeding period of 30 minutes. Fluorescence intensities were normalized to the level in wild type animals. Mean ± SEM is shown, n = number of worms analyzed. (PDF) [file pone.0070087.s006.pdf]

A

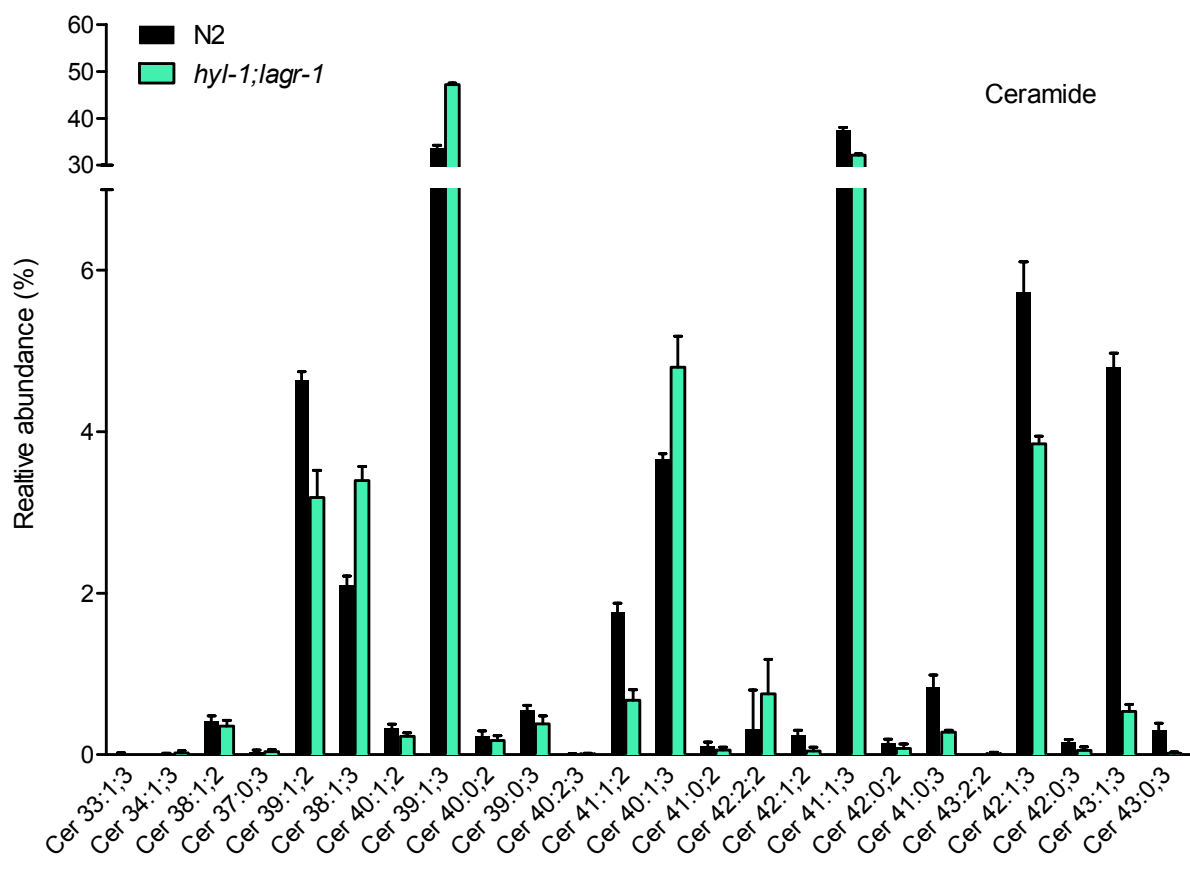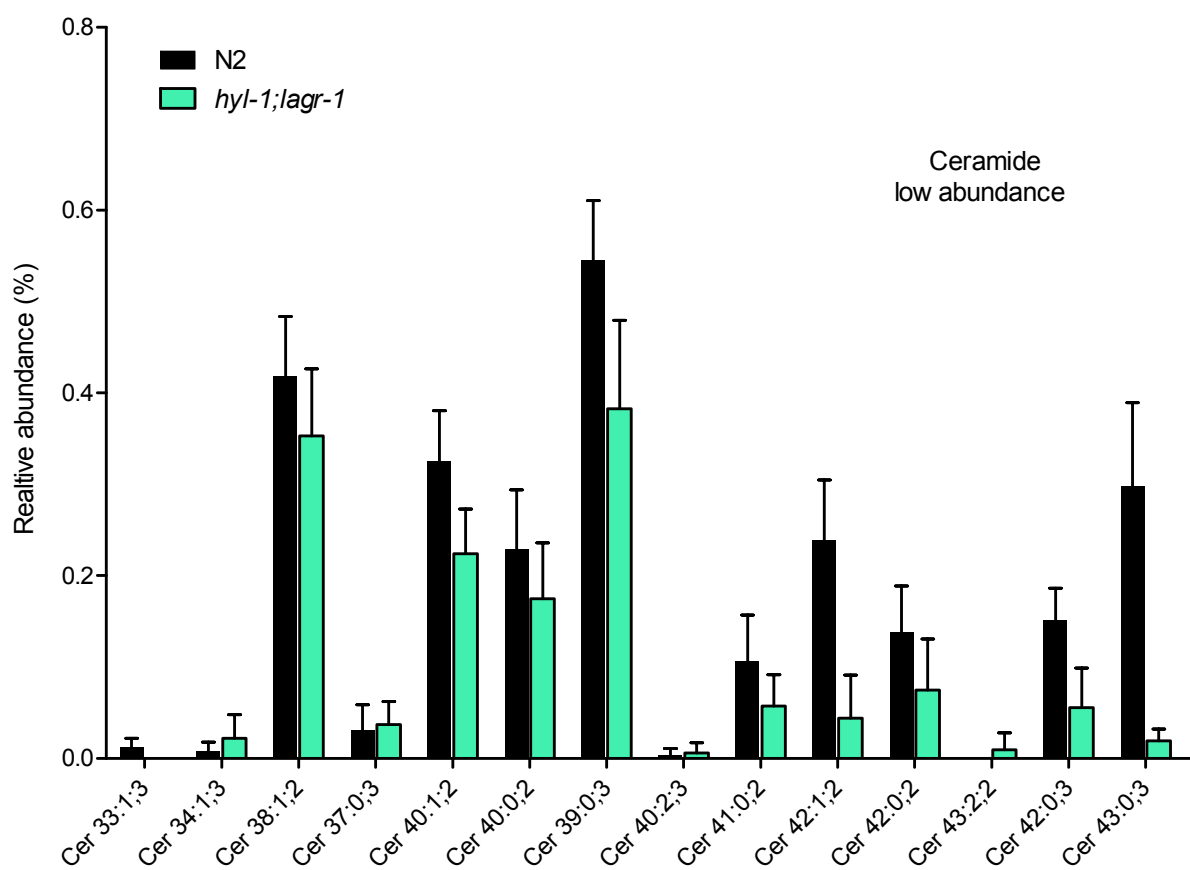

**B**

■ N2  
■ hyl-1;lagr-1

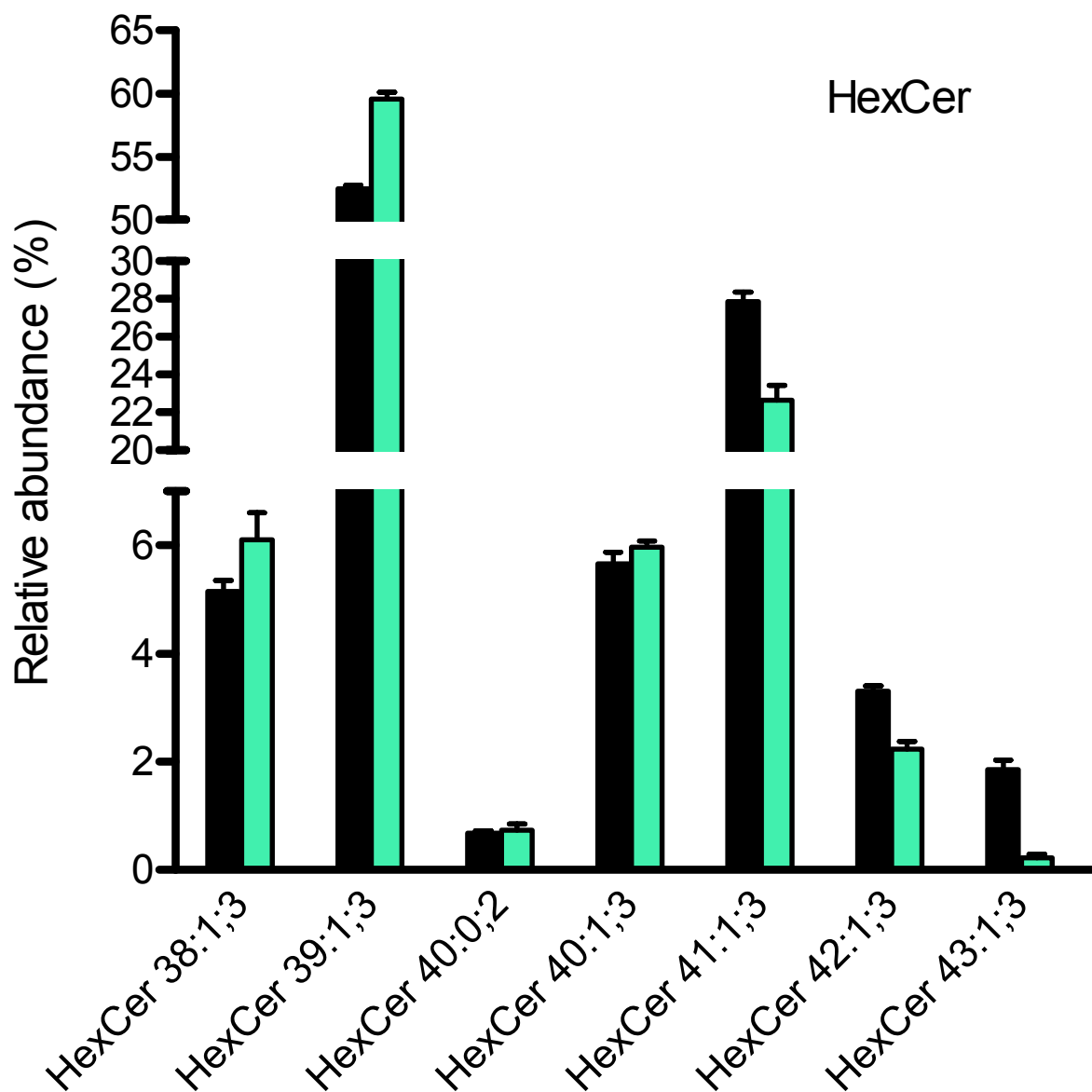

C

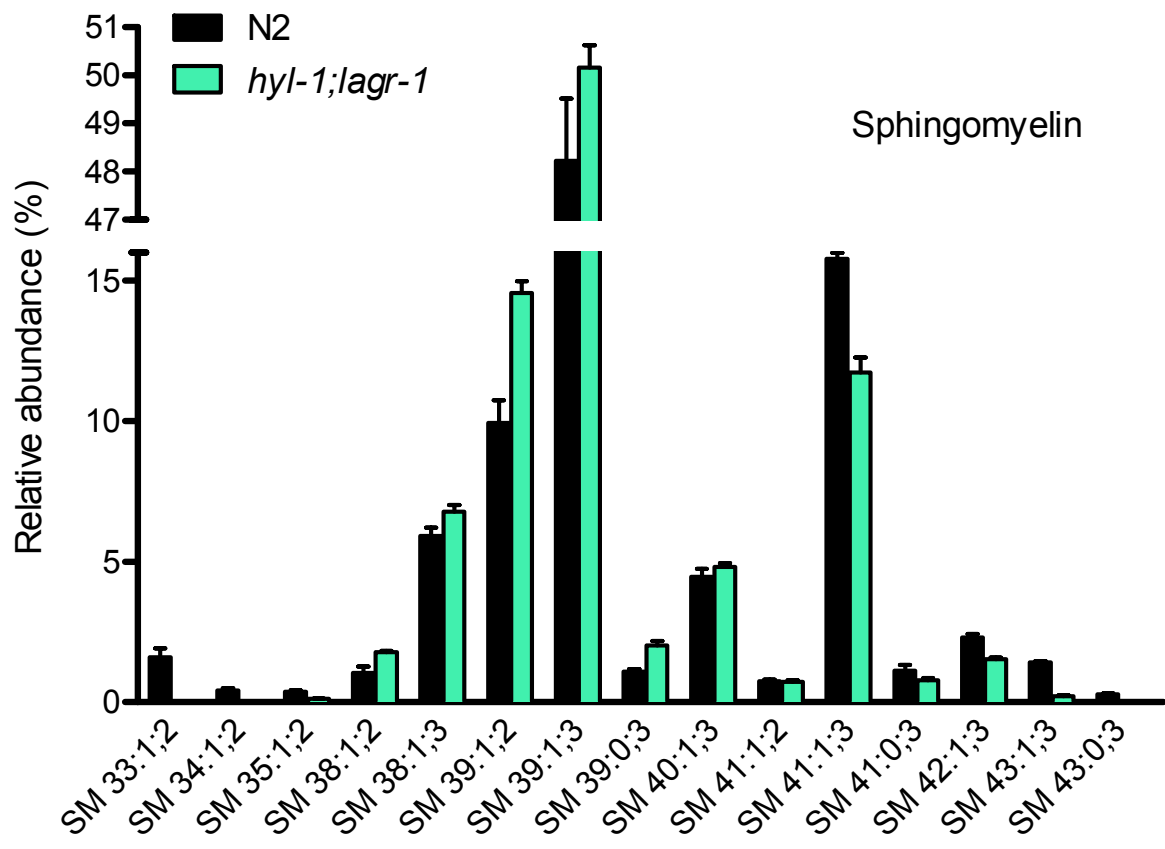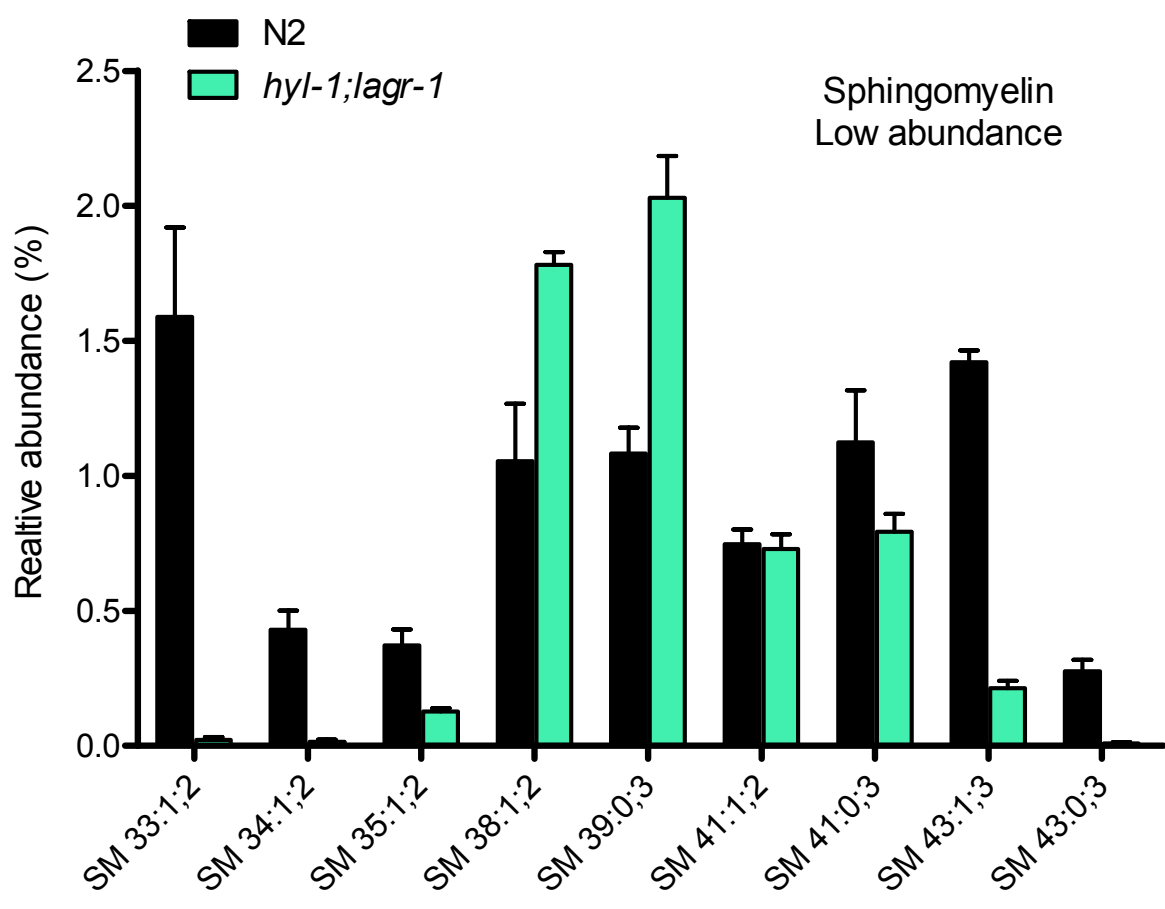

Supplement: Figure S7 — Overview of all Cer, HexCer, and SM species detected in hyl-1;lagr-1 . (A) Top: Relative abundance of all Cer species detected in hyl-1;lagr-1. Bottom: Zoom of low abundance Cer species detected in hyl-1;lagr-1. (B) Relative levels of all HexCer species detected in hyl-1;lagr-1. (C) Top: Relative abundance of all SM species detected in hyl-1;lagr-1. Bottom: Zoom of low abundance SM species detected in hyl-1;lagr-1. (PDF) [file pone.0070087.s007.pdf]

A

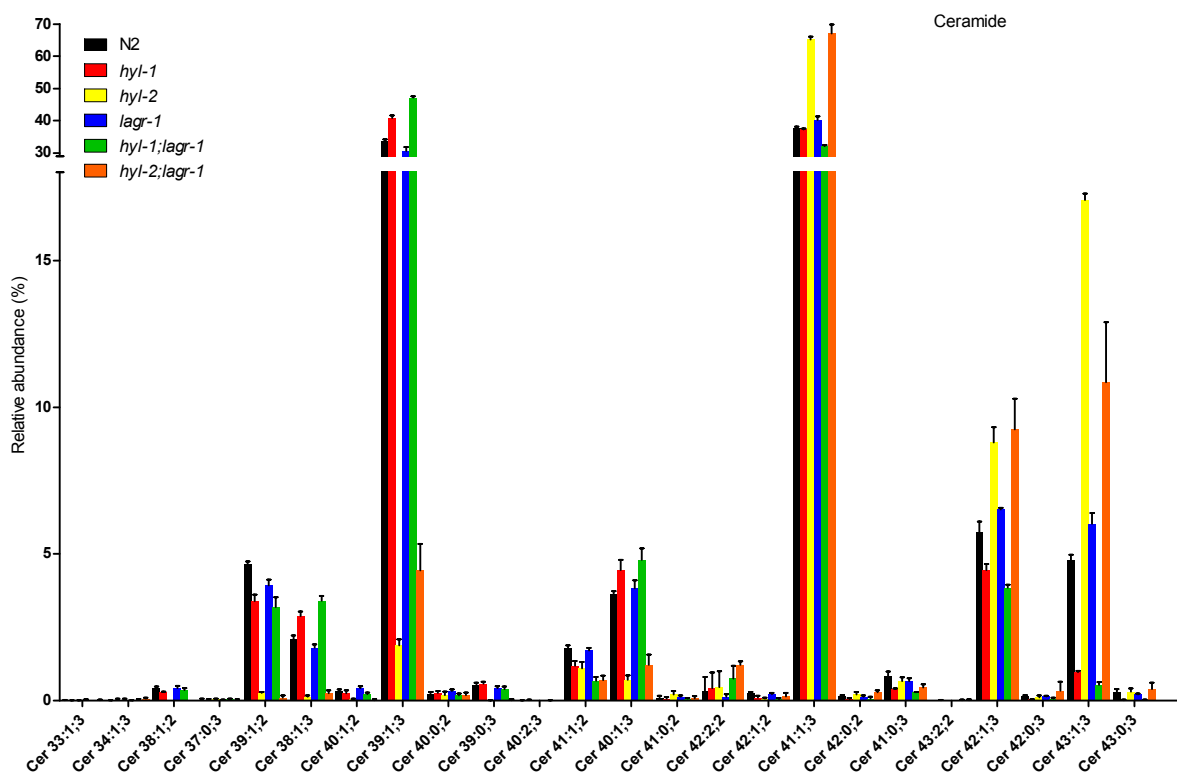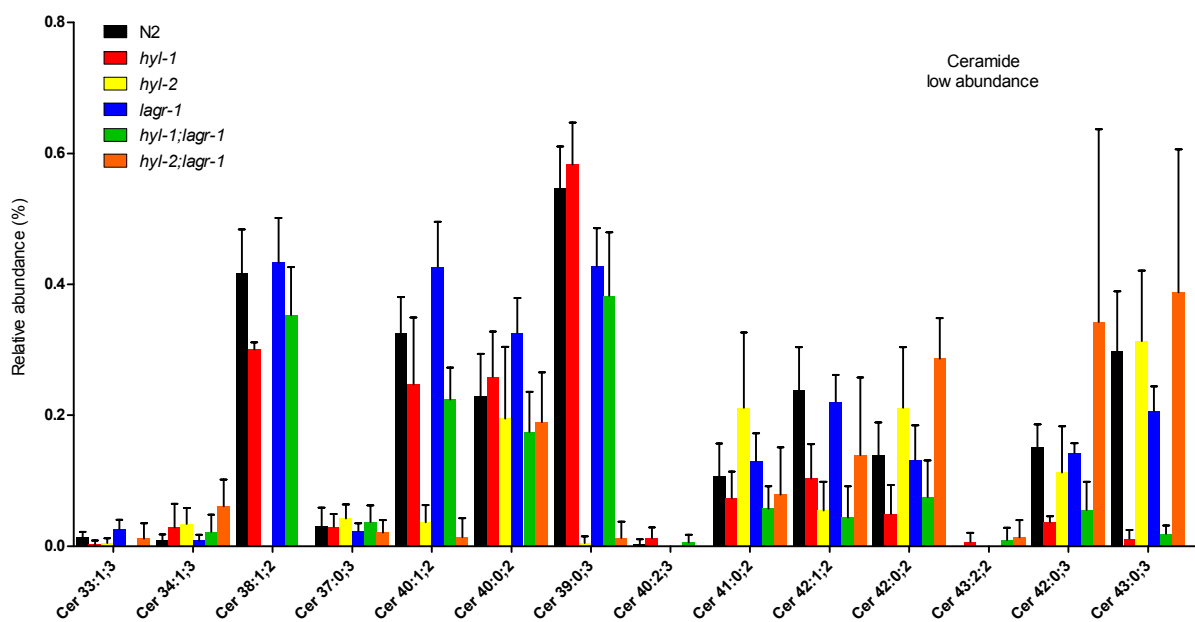

**B**

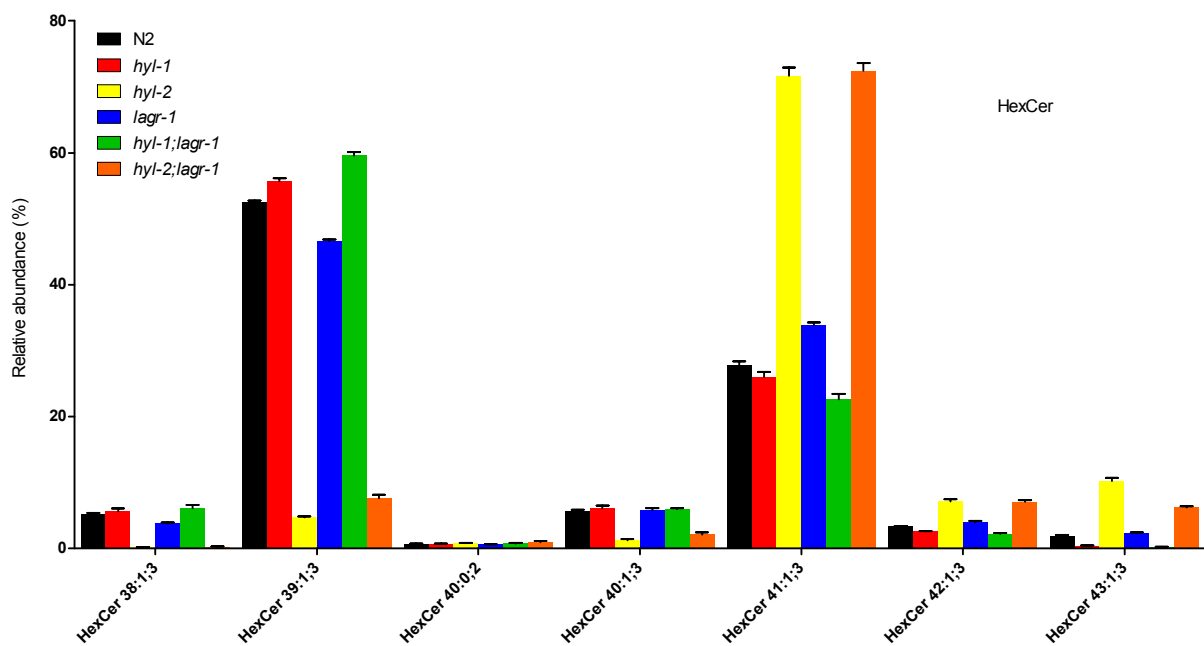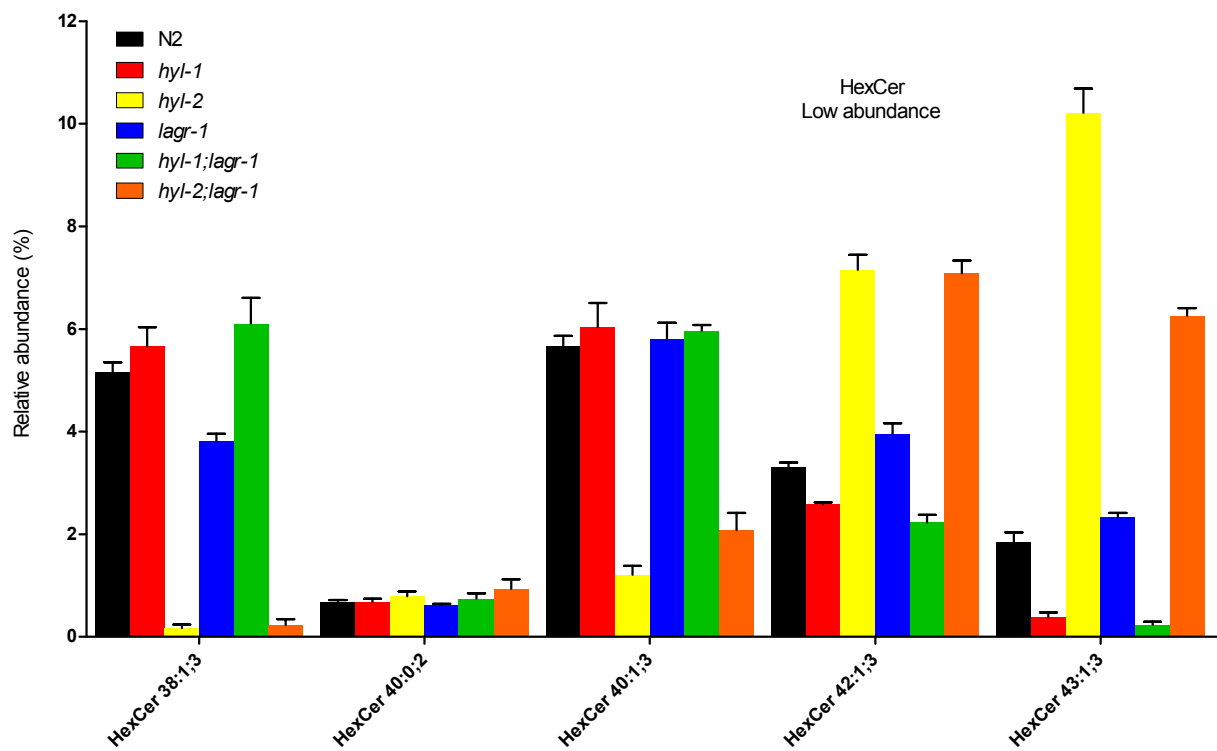

C

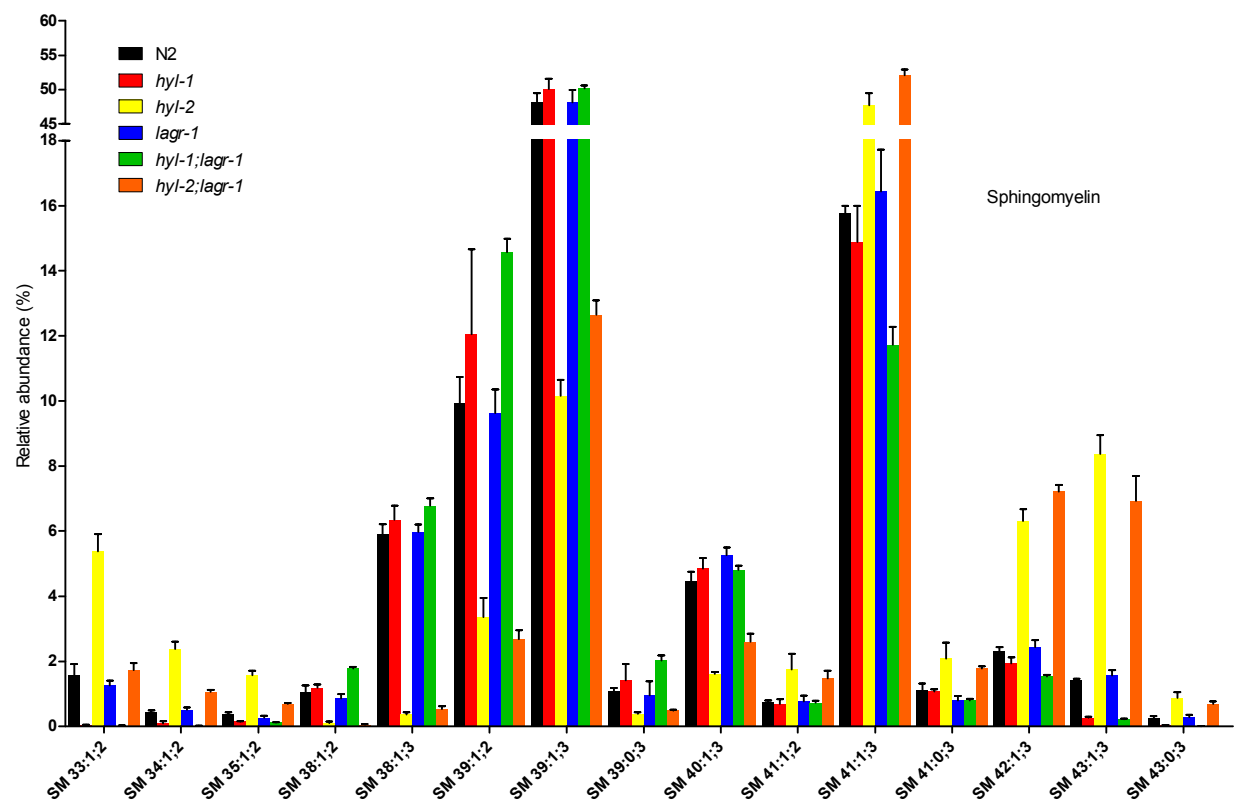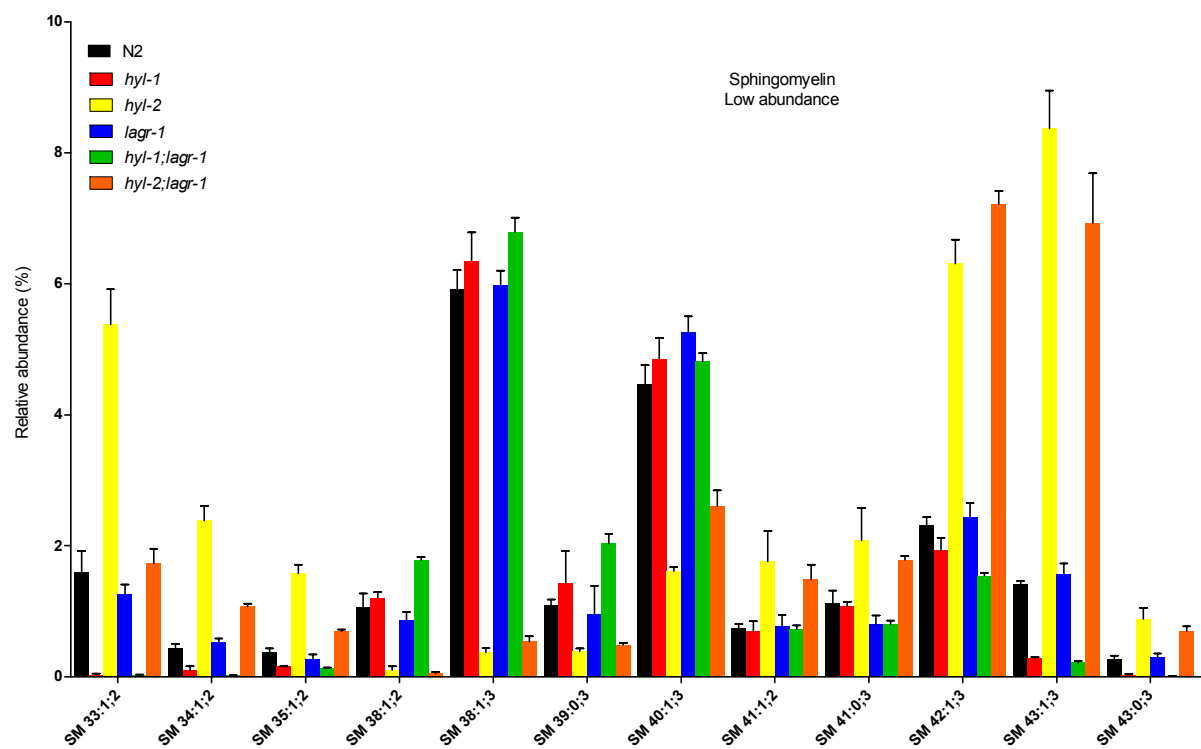

D

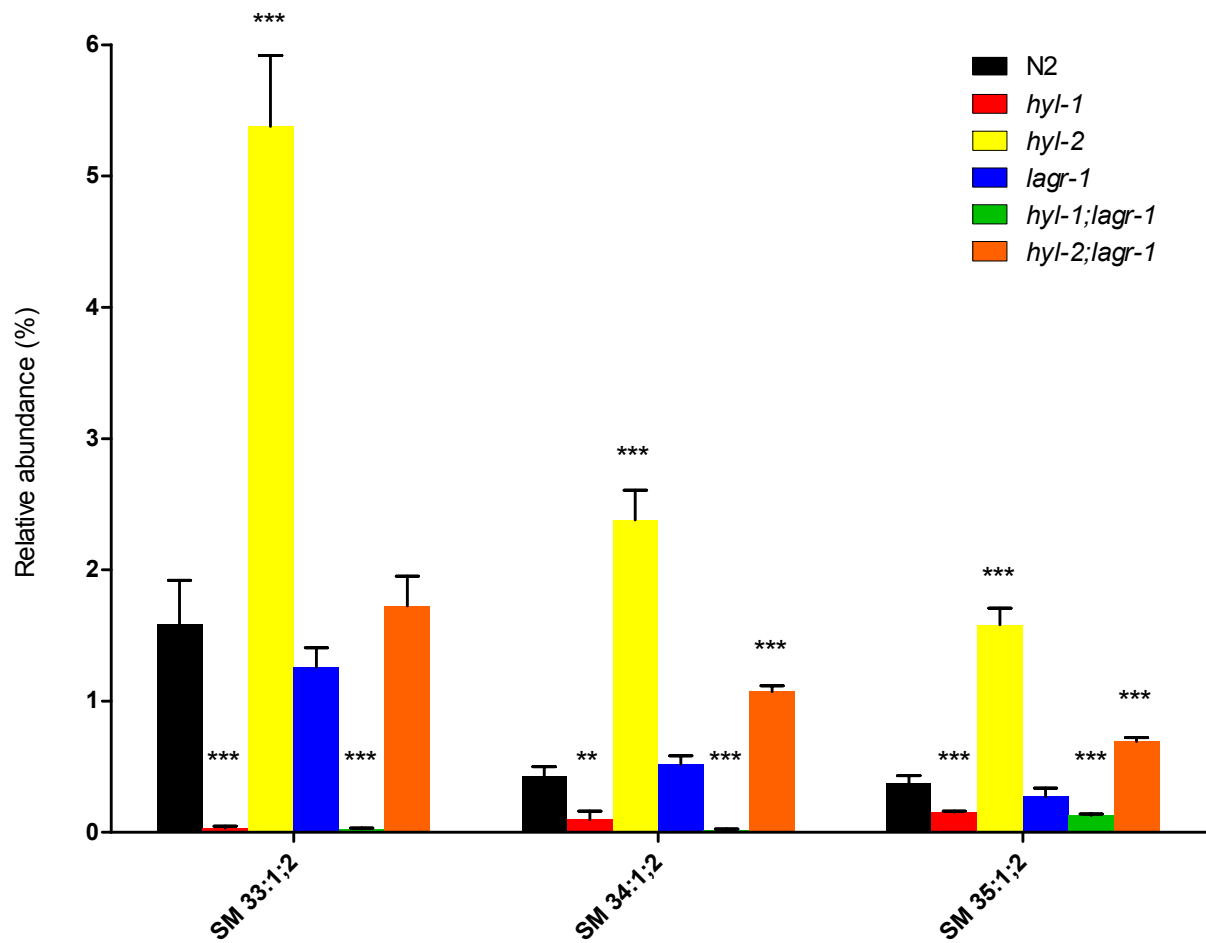

E

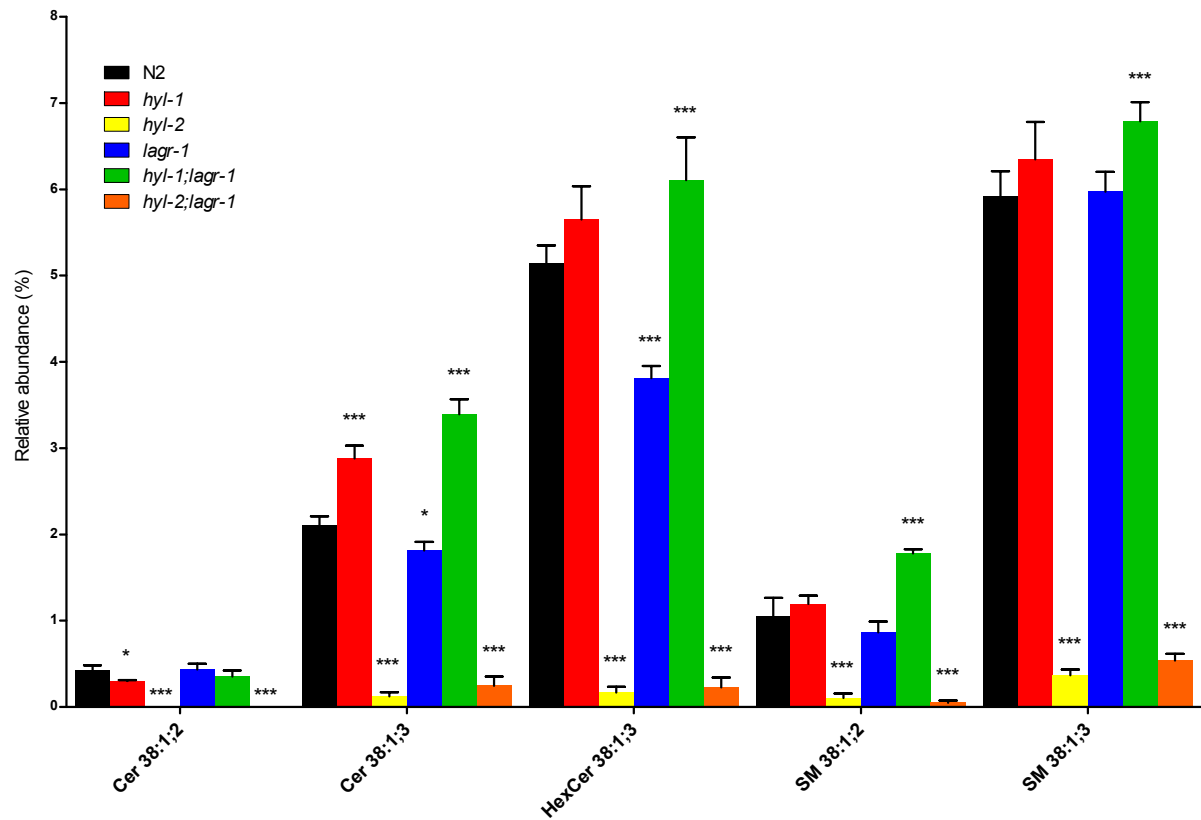

F

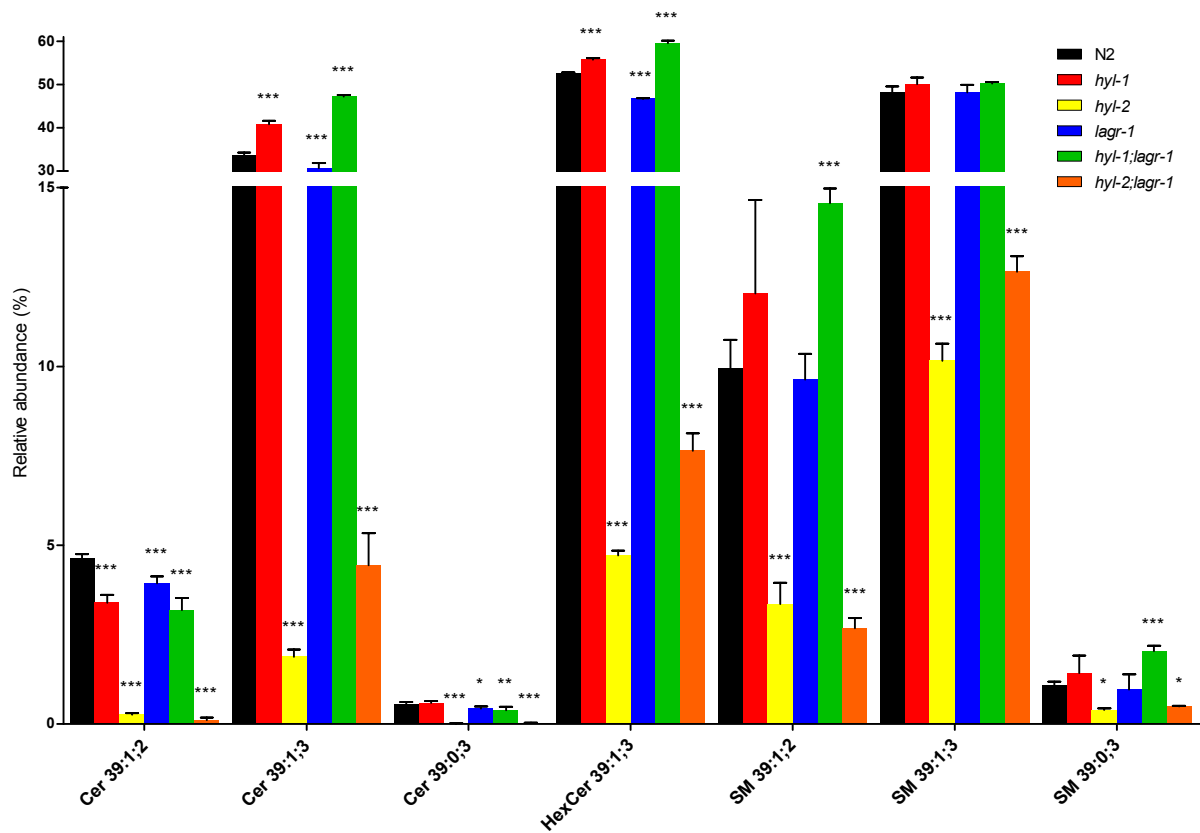

G

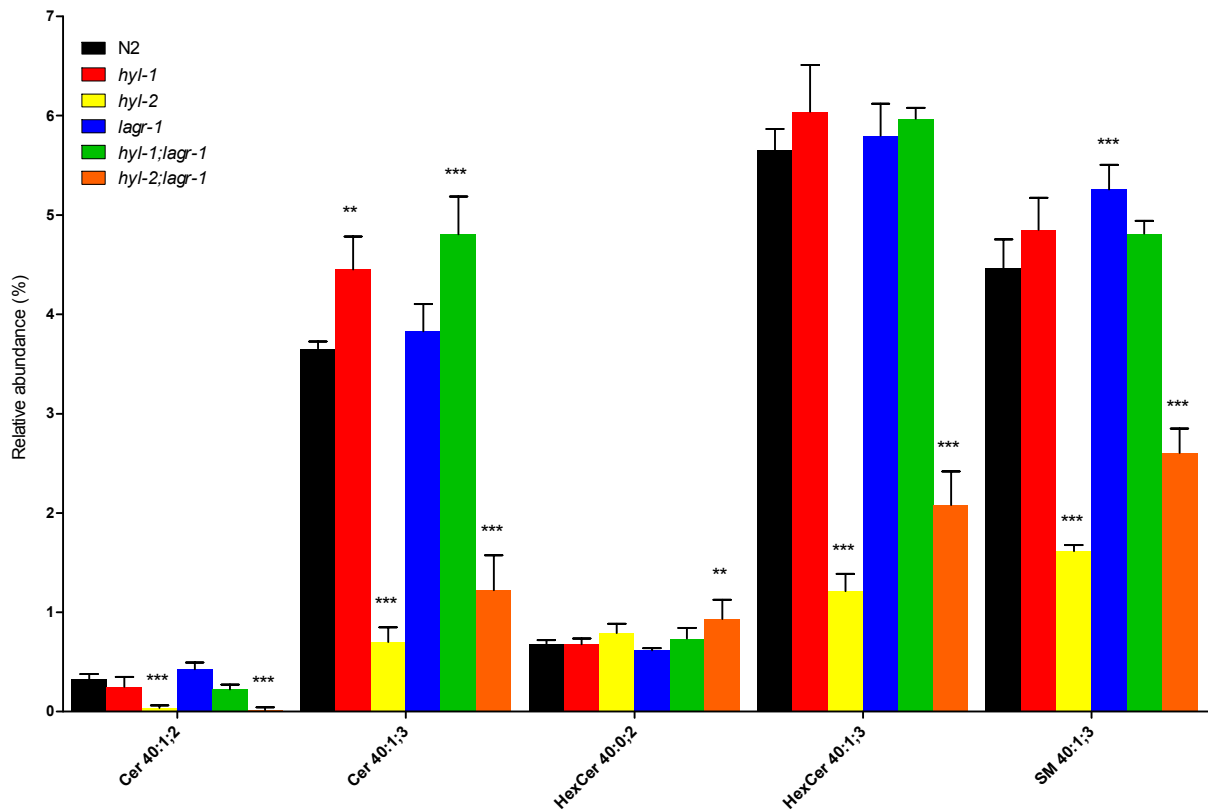

H

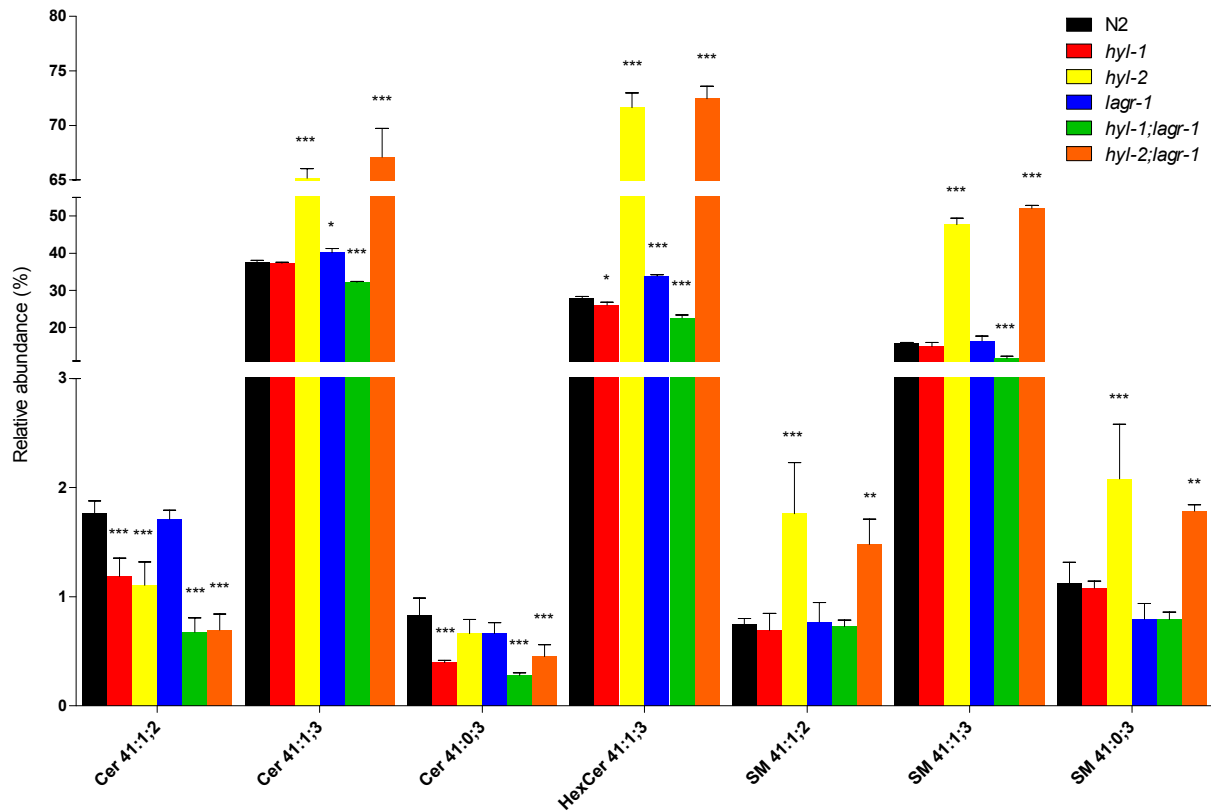

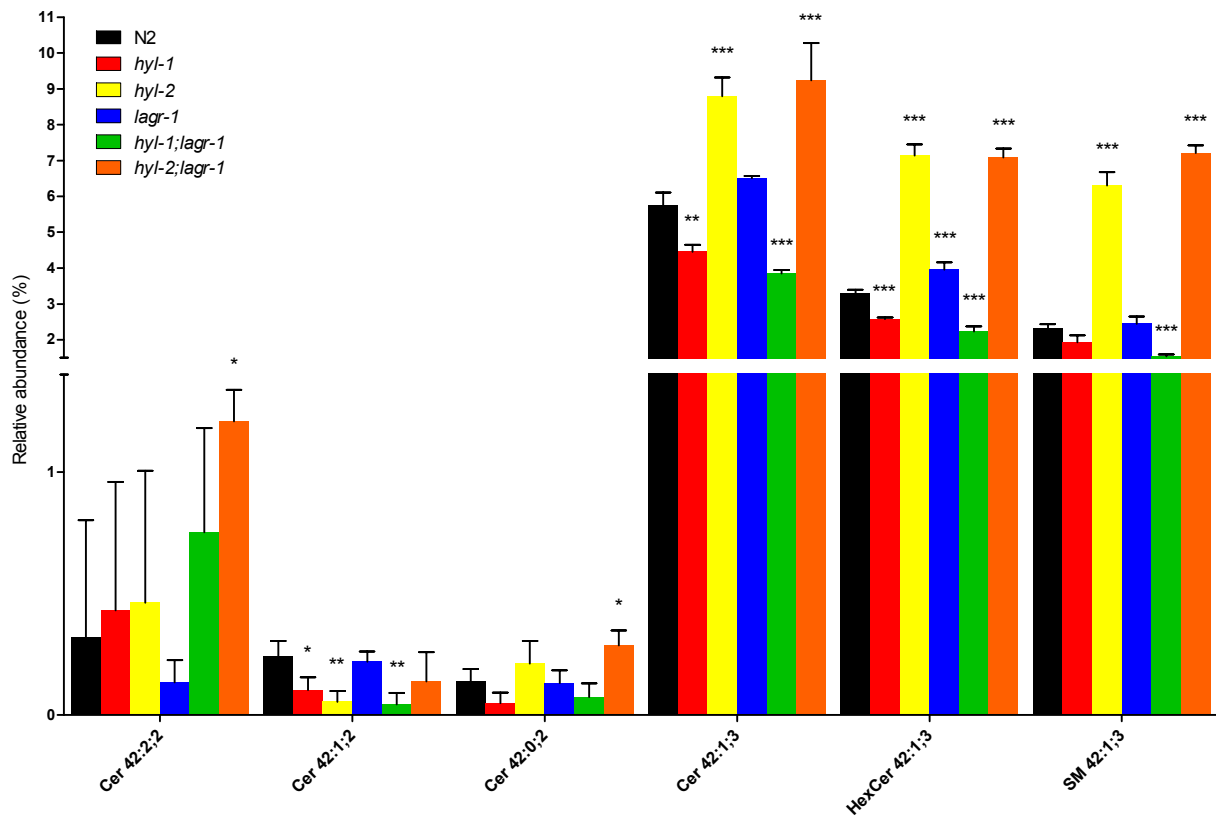

J

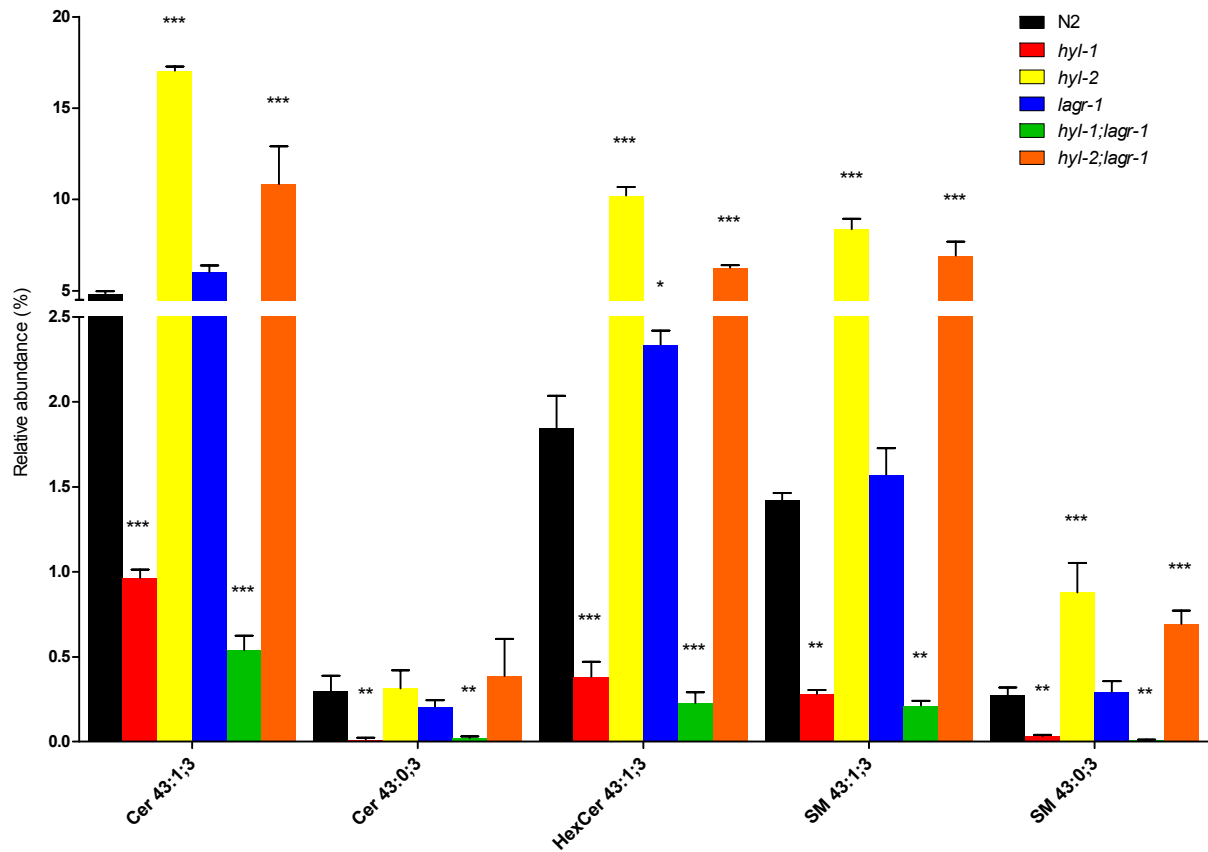

K

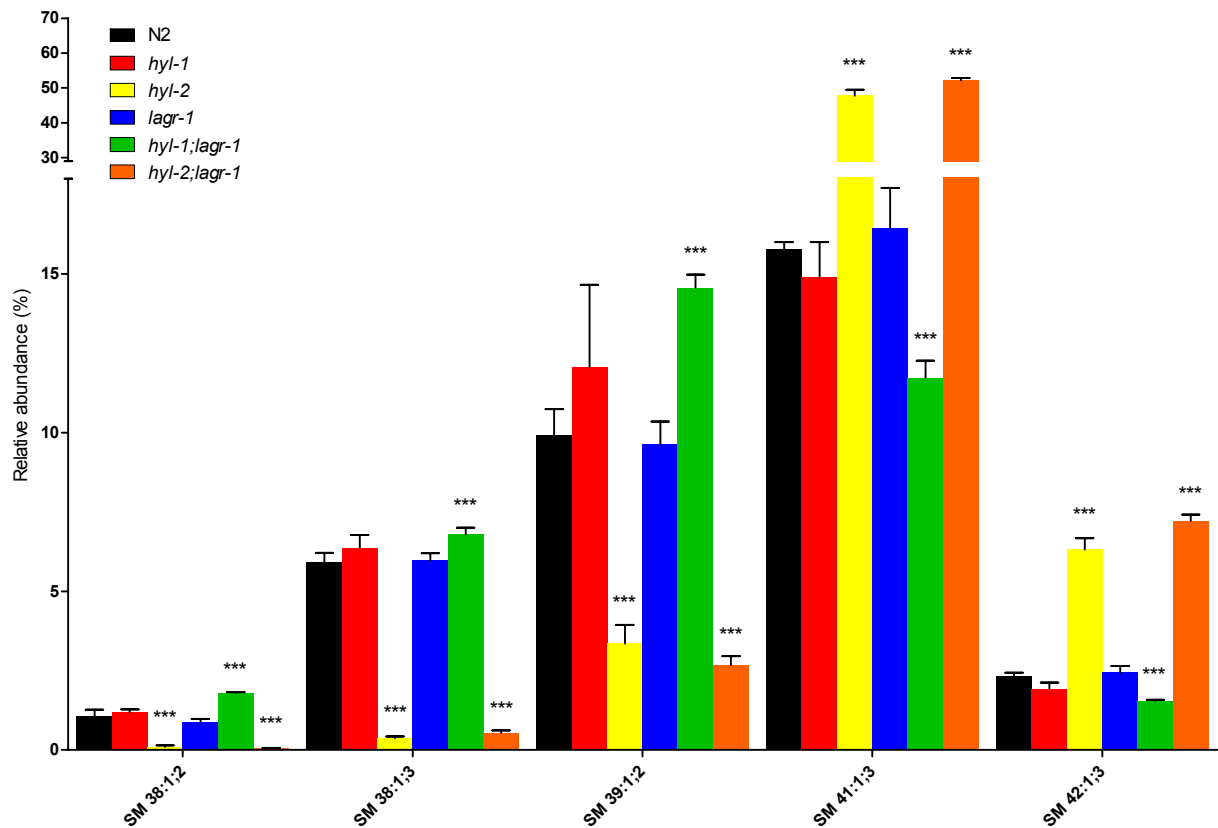

Supplement: Figure S8 — Overview of all Cer, HexCer, and SM species detected in the five different ceramide synthase mutants. (A) Top: abundance levels of all Cer species detected in ceramide synthase mutants. Bottom: Zoom of low abundance Cer species detected in ceramide synthase mutants. (B) Top: Relative levels of all HexCer species detected in ceramide synthase mutants. Bottom: Zoom of low abundance HexCer species detected in ceramide synthase mutants. (C) Top: Relative levels of all SM species detected in ceramide synthase mutants. Bottom: Zoom of low abundance SM species detected in ceramide synthase mutants. (D) Relative levels of all C33, C34, and C35 sphingolipid species detected to have significantly different levels in one or more ceramide synthase mutant compared to N2. (E) Relative levels of all C38 sphingolipid species detected to have significantly different levels in one or more ceramide synthase mutant compared to N2. (F) Relative levels of all C39 sphingolipid species detected to have significantly different levels in one or more ceramide synthase mutant compared to N2. (G) Relative abundance of all C40 sphingolipid species detected to have significantly different levels in one or more ceramide synthase mutant compared to N2. (H) Relative levels of all C41 sphingolipid species detected to have significantly different levels in one or more ceramide synthase mutant compared to N2. (I) Relative levels of all C42 sphingolipid species detected to have significantly different levels in one or more ceramide synthase mutant compared to N2. (J) Relative levels of all C43 sphingolipid species detected to have significantly different levels in one or more ceramide synthase mutant compared to N2. (K) Relative levels of all species significantly altered according to lifespan changes (e.g. Oppositely regulated in long-lived and short-lived strains). Statistical analyses were performed by one way analysis of variance followed by Dunnett’s multiple comparisons test using GraphPad Prism v [file pone.0070087.s008.pdf]

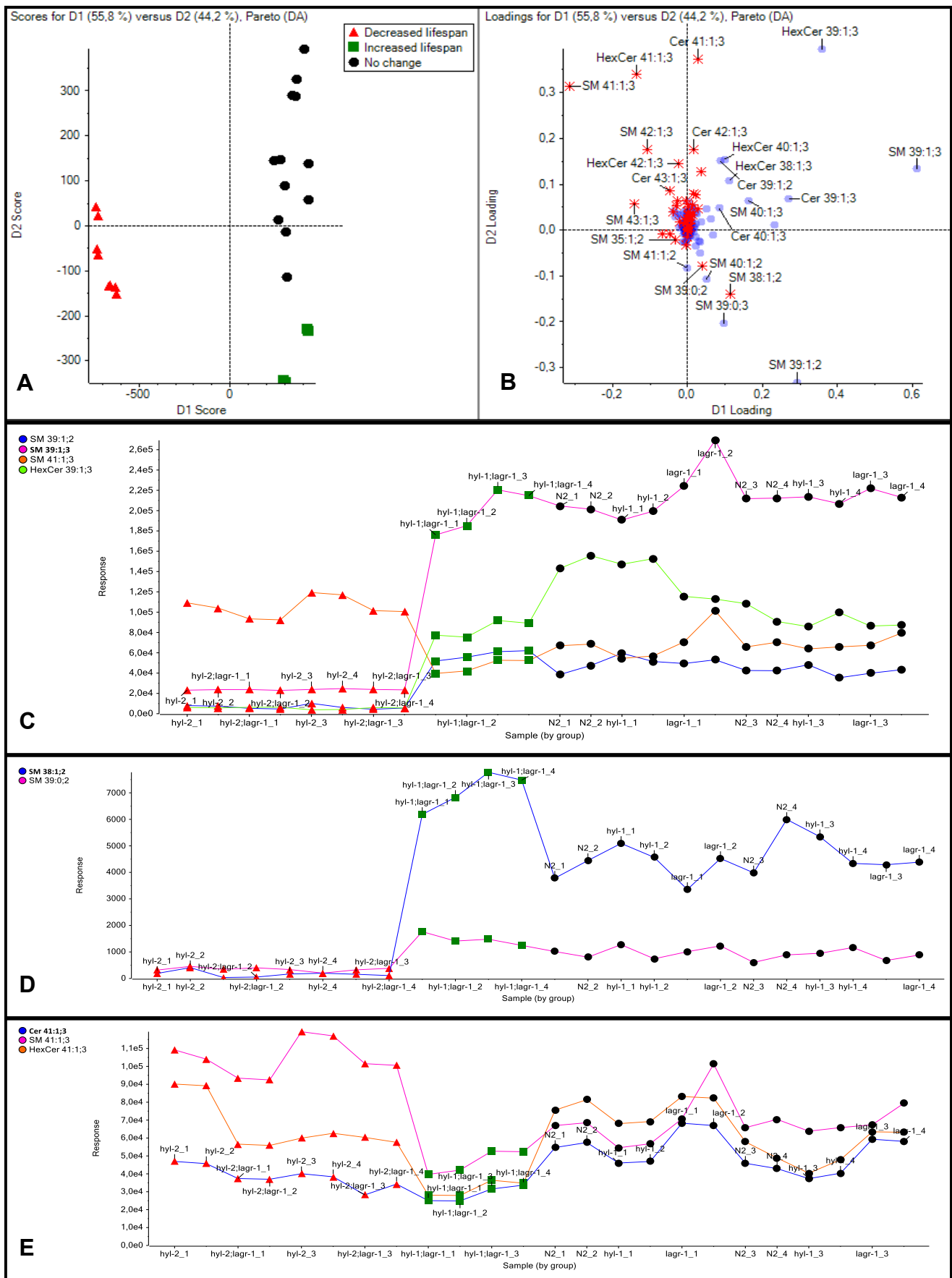

Supplement: Figure S9 — Multivariate analysis by principal component analysis of sphingolipidomic data segregated according to lifespan changes. (A) Score plot projecting the first (D1) and second (D2) principal components show a clear separation of the strains when grouped according to the following lifespan changes: “Decreased lifespan ”, “Increased lifespan ”, and “No change ”. D1 and D2 account for 100% of the total sample variance. (B) Loading plot depicting the sphingolipid species contributing the most to the total sample variance. Red stars denotes the species significantly altered when comparing hyl-1 (no lifespan change) to hyl-1;lagr-1(increased lifespan). (C) Relative abundance of the four species contributing the most to the score plot separation. (D and E) The 5 species which contribute most to the separation and are significantly altered in hyl-1;lagr-1 compared to hyl-1 (denoted by red stars in B). (PDF) [file pone.0070087.s009.pdf]

**A** *pHYL-1::GFP fusion*

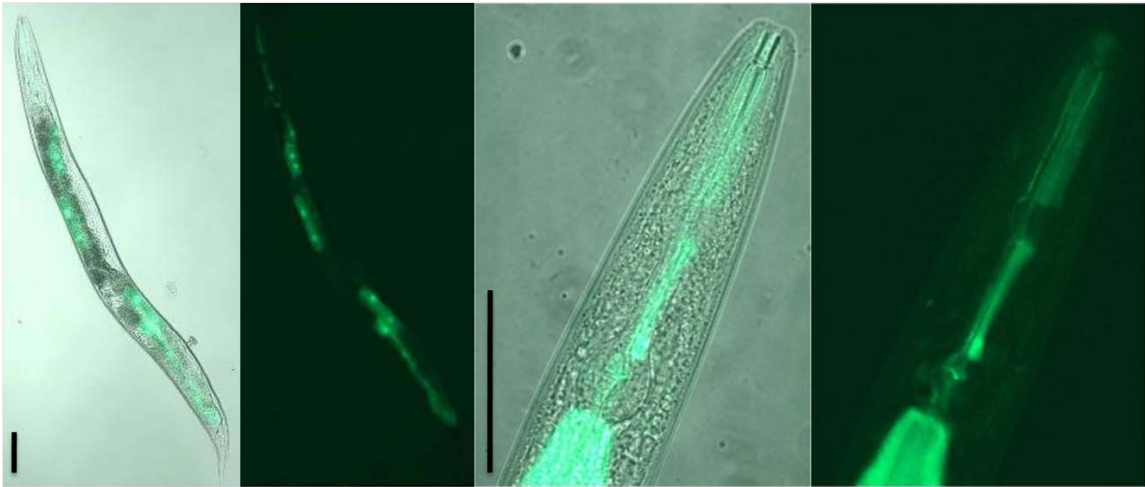

**B** *HYL-2::GFP fusion*

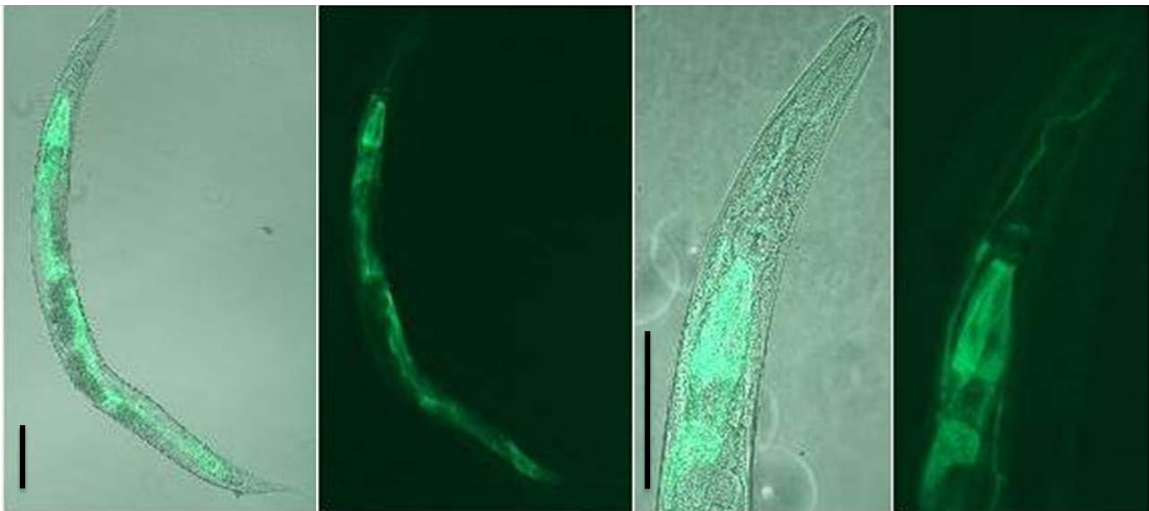

**C** *pLAGR-1::GFP fusion*

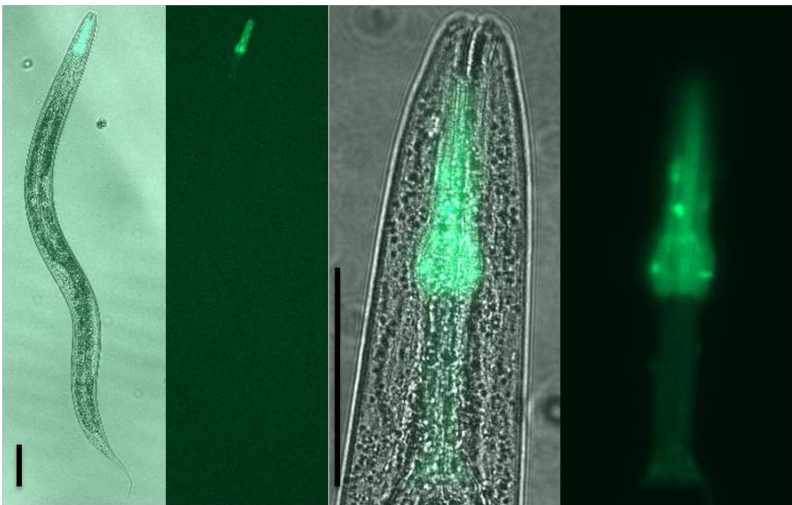

Supplement: Figure S10 — Expression patterns of the ceramide synthase genes at the L4 stage. (A) HYL-1 shows expression in the body wall muscles, the pharyngeal muscles PM3 and PM5, and unidentified cells in the pharynx. (B) HYL-2 shows expression in the body wall muscles and the nervous system. (C) LAGR-1 shows expression in the pharyngeal muscles PM3-5 and in pharyngeal nerves. Scale bar: 80 µm. (PDF) [file pone.0070087.s010.pdf]
