# Supplementary material for: Functional Loss of Two Ceramide Synthases Elicits Autophagy-Dependent Lifespan Extension in C. elegans
Source: PLoS One. 2013 Jul 19;8(7):e70087. doi: 10.1371/journal.pone.0070087 (PMC3716707; doi:10.1371/journal.pone.0070087)
Supplement: Figure S2 — Lifespan analyses of all ceramide synthase mutants. Cumulative lifespan analyses of N2, hyl-1, hyl-2, lagr-1, hyl-1;lagr-1, and hyl-2;lagr-1 grown on OP50 at 20°C. (A) hyl-1 displays no change in lifespan compared to N2 (P = 0.3592). (B) hyl-2 displays a shortened lifespan compared to N2 (P<0.0001). (C) lagr-1 displays no change in lifespan compared to N2 (P = 0.1651). (D) hyl-1;lagr-1 displays an extended lifespan compared to N2 (P<0.0001). (E) hyl-2;lagr-1 displays shortened lifespan compared to N2 (P<0.0001). (F) Table summarizing the depicted lifespan analyses. aSome animals were censored as they crawled of the plate, ruptured, or died as a “bag of worms”, however they are incorporated in the data set up until the day they were censored. The number of individual trials is in parentheses. Statistical analyses were performed by Gehan-Breslow-Wilcoxon tests using GraphPad Prism version 6.0 (GraphPad Software). bThe Bonferroni method was used to correct for multiple comparisons and P-values below 0.01 are considered statistically significant equivalent to a significance level of 0.05. Cumulative statistics is shown in this table as experimental animals subjected to the same treatment behaved similarly between trials. (PDF) [file pone.0070087.s002.pdf]

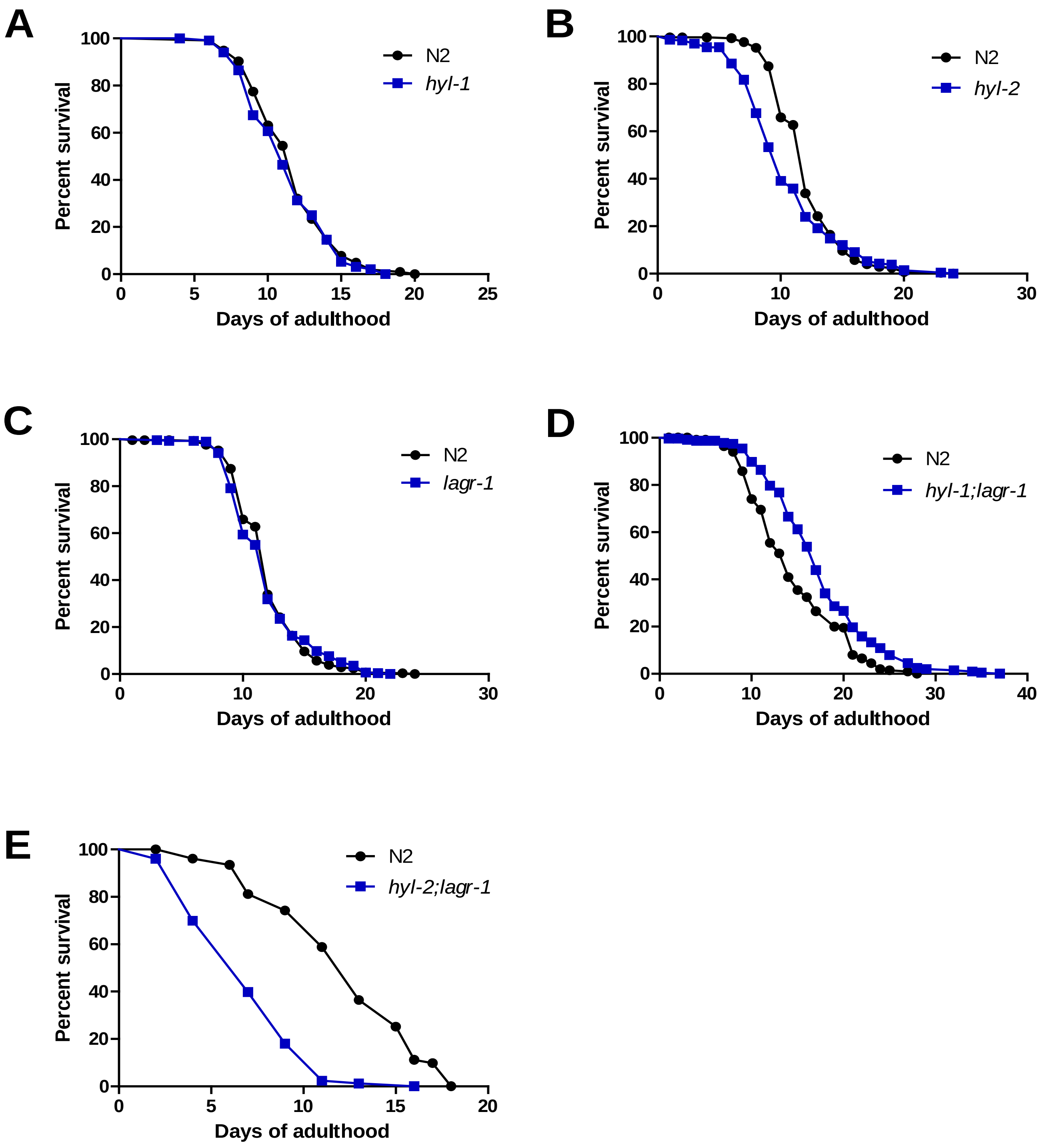

F

Strain	Adult Lifespan	Number of Animals[a]	Control Lifespan	Number of Control Animals	Percent Lifespan Change	*p*-Value Versus Control[b]
hyl-1	11	101/120 (1)	12	105/120 (1)	-8.3%	0.3592
hyl-2	10	224/300 (2)	12	282/300 (2)	-16.7%	<0.0001
lagr-1	12	278/300 (2)	12	282/300 (2)	0.0%	0.1651
hyl-1;lagr-1	17	205/240 (2)	14	202/240 (2)	21.4%	<0.0001
hyl-2;lagr-1	7	87/100 (1)	13	72/80 (1)	-46.2%	<0.0001
